# Discrimination of entangled photon pair from classical photons by de Broglie wavelength

**DOI:** 10.1038/s41598-020-63833-8

**Published:** 2020-04-27

**Authors:** Valentin Mitev, Laurent Balet, Nicolas Torcheboeuf, Philippe Renevey, Dmitri L. Boiko

**Affiliations:** CSEM, rue de l’Observatoire 58, Neuchâtel, 2000 Switzerland

**Keywords:** Optics and photonics, Physics

## Abstract

Quantum optics largely relies on the fundamental concept that the diffraction and interference patterns of a multi-partite state are determined by its de Broglie wavelength. In this paper we show that this is still true for a mixed state with one sub-system being in a classical coherent state and one being in entangled state. We demonstrate the quantum-classical light discrimination using de Broglie wavelength for the states with all classical parameters being the same.

## Introduction

The multiphoton quantum states keep the promise of growing importance in nowadays and future practical applications. This is stemming from the fact that the entangled N photons of wavelength λ propagate as a solitary entity at the de Broglie wavelength of λ_N_ = λ/N. This can be detected provided the detectors are properly arranged in the experimental setup^1,2^. One example is the optical lithography with entangled photons^3,4^ promising to achieve higher component density in the microelectronics devises. Imaging with non-classical photons, allowing one to bypass the Rayleigh resolution limit and classical shot-noise level, is another notable example^5–7^.

The practical schemes realizing quantum imaging (or lithography) are expected to operate with sources having high production rates of correlated photons^8–10^. However, non-ideality of sources capable to produce multi-partite photon states as well as optical losses destroying entanglement may result in mixed states, where the entangled and classical photons have the same wavelength, polarization and propagation direction. This makes impossible to discriminate them by using any of the classical variables such as the optical wavelength, polarization etc. On the other hand, if such mixed entangled and classical state is incident in the same beam on the optical detector, as it will be the case in a quantum imaging setup, this will compromise the fidelity and purity of the detected quantum state or even lead to the detector saturation and just a small fraction of entangled photons being recorded.

Thus one important step towards realizing quantum imaging with high photon rate in the presence of spurious light background will be a separation of non-classical photon states from the classical ones.

It has been already reported^11,12^ that under certain conditions, the quantum diffraction of bi-photons at a grating manifests a single-point second-order correlation function *G*^*(2)*^*(k,k)* with the maxima at the diffraction lobes in the first-order (intensity) pattern *G*^*(1)*^(2*k*) of classical photons at a half wavelength λ/2. The entangled photon pair of the wavelength λ has the de Broglie wavelength of λ_DB_ = λ/2 that defines such second-order diffraction pattern and, hence, the physical presence of bi-photons in the respective diffraction angles of the configuration space. This observation indicates that photon pairs are physically present in each of these directions, which is the base for our concept to discriminate the quantum and classical states of the same wavelength by a quantum diffraction or quantum interference. In this work we implement this concept using quantum diffraction on an echelle grating and demonstrate the quantum-classical light discrimination (QCD) by diffraction at de Broglie wavelength.

## Experimental set-up

**Figure 1:**
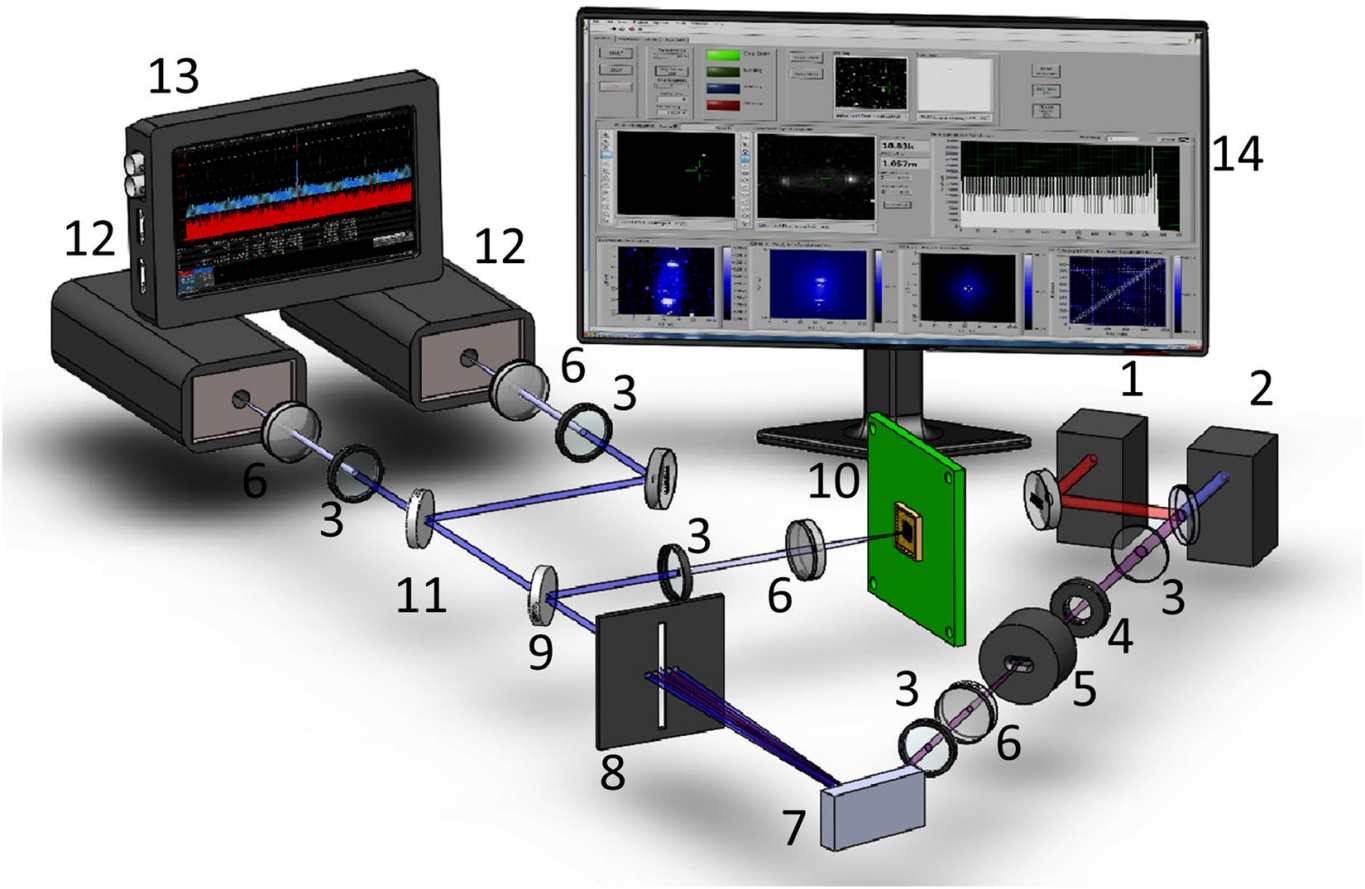
Experimental setup: 1. Laser at 795 nm; 2. Laser at 405 nm; 3 Lenses (different in the different positions); 4. Half-wave plate; 5. PPKTP crystal; 6. Filters (different for the different positions and arranged beams combinations); 7. Echelle grating; 8. Adjustable slit; 9. Flip-flop mirror, selecting respectively spatial and temporal correlation measurements; 10. SuperEllen SPAD-array detector; 11. Beamsplitter; 12. Standalone single-SPAD detector modules. 13. Digital oscilloscope; 14. PC interface of SuperEllen detector. Note: Not numbered optical elements are mirrors.

The setup used for demonstration of the QCD concept is presented in Fig. 1. It consists of four main parts: the mixed-state photon source, quantum-classical discriminator and two coincidence detection chains for recording, respectively, the time-difference *G*^*(2)*^(*t-t*′) or spatial *G*^*(2)*^(***k***, ***k***′) correlation patterns in the far-field of the echelle grating.

The dedicated source of mixed photon states is built around a traditional entangled photon source based on a type-0 spontaneous parametric down-conversion (SPDC) in a periodically poled potassium titanyl phosphate (PPKTP) crystal^13,14^. The crystal is cw pumped by a volume Bragg grating (VBG) stabilized GaN diode laser^15^ at 405 nm wavelength and produces the signal-idler photon pairs at 810 nm. After the PPKTP crystal, the light is a mixed state of classical coherent state at 405 nm and a quantum bi-photon state at 810 nm. When needed, two optical filters block the classical light at 405 nm by 120 dB attenuation. On the optical axis of the pump laser and the SPDC source we add a low-noise single transverse and polarization mode VCSEL laser beam at 795 nm. This VCSEL laser is an independent source of highly coherent classical state at the wavelength close to the optical one of the bi-photon state. The wavelength of the coherent state source is intentionally detuned from 810 nm to exclude possibility of parametric amplification in the PPKTP crystal^16,17^. In addition, the power in the coherent state beam is reduced to the power level of the SPDC bi-photon source (~6 nW) to avoid detector saturation.

The quantum-classical photon state discriminator (QCD) is implemented using an echelle grating (Thorlabs GE2550-0363 with 31.6 groves /mm) followed by an adjustable vertical slit located at a specific diffraction order (see below). With sufficiently open slit we may observe several consecutive diffraction orders. Alternatively, by precisely positioning the slit and adjusting its width, we may select only one diffraction order, which is classically prohibited at the given optical wavelength. We demonstrate QCD operation with incident probe beam containing classical and entangled photons at a choice, by properly adjusting a combination of cut-off and band-pass filters.

The spatial four-dimensional (4D) *G*^*(2)*^*(****k***, ***k***′) correlation patterns at the QCD output are captured with a novelty 32 × 32 pixels single-photon avalanche diode (SPAD) array detector SuperEllen^18–20^. It is based on time-to-digital converter (TDC) architecture and enables 160 ps resolution in the coincidence detection. In order to accommodate for possible TDC delays and jitter across the array, the coincidence resolution is put to 480 ps. The Photon Detection Probability (PDB) is >20% at 420 nm and >5% at 800 nm. More details about the detector and its functions as well as data processing and representation of correlation patterns are given in Supplementary Information. The sensitive surface of the array detector is placed at the back focal plane of the objective lens, thus detecting the far-field (FF) spatial correlation patterns. The imperfections of such SPAD arrays are linked to their relatively high pixel dark count rates and neighboring pixel crosstalk^21,22^, which, respectively, upraise the accidental coincidence background due to events that are uncorrelated by nature and produce the false positive correlation signatures. Here we report spatial correlation patterns *G*^*(2*)^*(****k***, ***k***′) obtained after a correction to reduce such crosstalk and accidental coincidences background ~*G*^*(1*)^*(****k***) × *G*^*(1)*^*(****k***′*)*. (The correction procedure is detailed in the Supplementary Information.) To further avoid possible ambiguities caused by residue crosstalk signatures, we perform the spatial correlation measurements using non-collinear SPDC regime^14^, so as the signal and idler photons hit non-neighboring pixels in the detector array and reveal negative (anti-) correlation traces.

The time-difference *G*^*(2)*^*(t-t*′*)* correlation functions are recorded using two standalone single-SPAD detector modules (ID Quantique id100-50) placed at the output ports of a beamsplitter (BS). The SPAD outputs are recorded on a digital oscilloscope (Teledyne LeCroy SDA 8137-B). The same oscilloscope also builds the start-stop histograms of delayed photon arrivals. This coincidence detection channel does not suffer from detector crosstalk. We report on QCD operation for both collinear (frequency non-degenerate) and non-collinear (degenerate) SPDC regimes^14^, attesting that non-collinearity of the correlated photon pair has no impact on QCD operation. In the Supplementary Information we experimentally and theoretically show that there is no requirement for corelated photon pair being localized within a spot of the size smaller than the feature to be resolved^3,4^. In our case this is the echelle grating pitch used in the QCD. The only requirement is about the uncertainty of mutual locations of the partite composing the bi-photon state, namely, about its correlation width to be smaller than the grating pitch.

## Quantum and classical diffraction regimes

**Figure 2:**
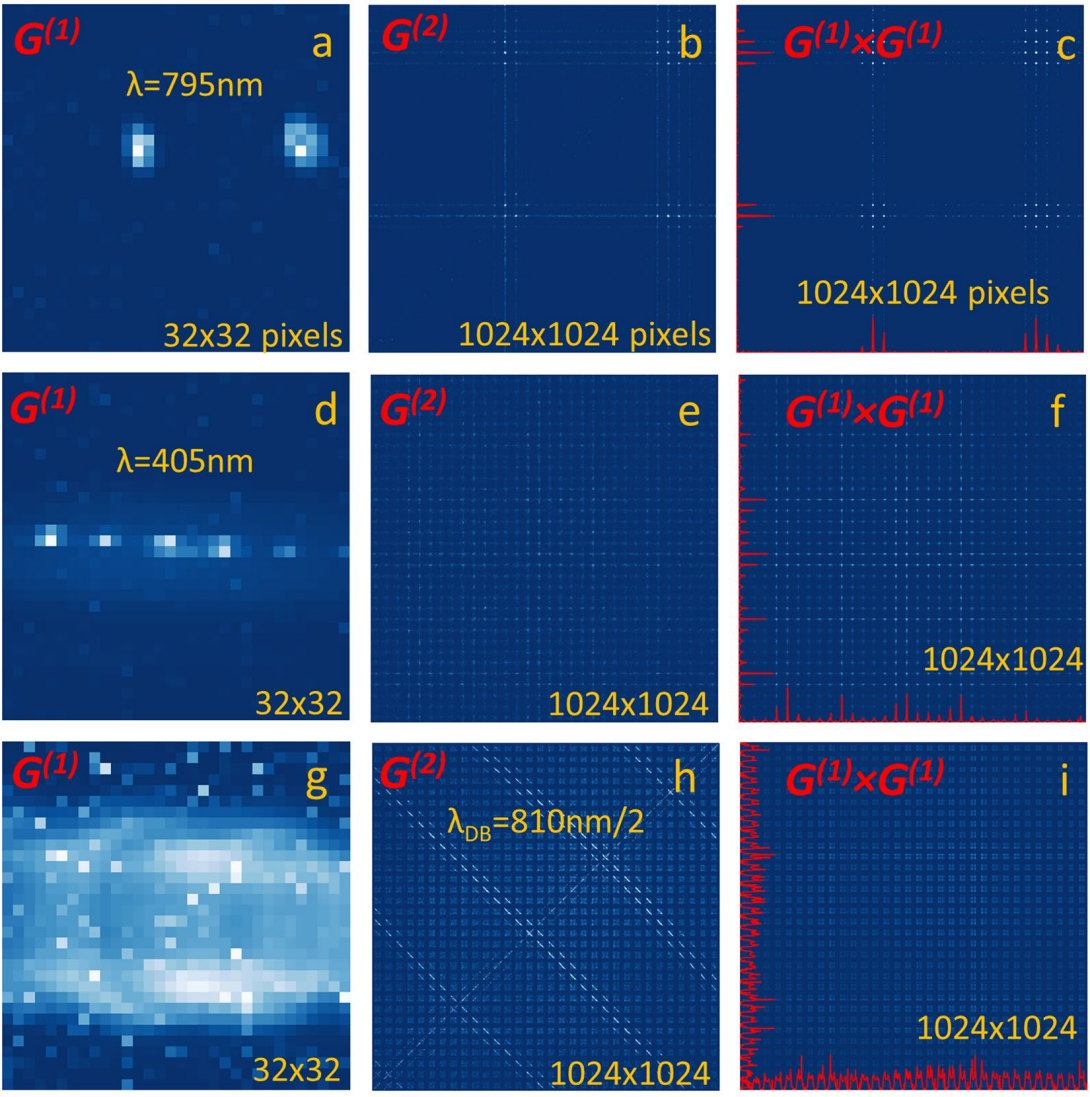
First- and second-order correlation patterns measured in the far field of echelle diffraction grating illuminated with various pure states (coherent and bi-photon states). Top row panels depict two successive diffraction orders of coherent state at 795 nm from VCSEL, showing (a) the intensity distribution G^(1)^(k_x_,k_y_), (b) second-order correlation pattern G^(2)^(k,k′) after pixel crosstalk correction and accidental removal represented as 2D map spanned over linearized indexes of the two array pixels and (**c**) its classical counterpart G^(1)^(k) × G^(1)^(k′) due to accidental coincidences. Middle row panels (d–f) show similar patterns for five successive diffraction orders of 405 nm pimping laser. Bottom row panels (g–i) display similar patterns for five successive two-photon diffraction orders of bi-photon state produced in non-collinear SPDC regime at 810 nm wavelength. The residue correlations seen as vertical lines in (b,e) are due to photon detection in one of the diffraction lobe and spurious light or dark count at any other pixel of the array.

Figure 2(a,d) show the intensity diffraction patterns *G*^*(1)*^(*k*_*x*_, *k*_*y*_) for the pure coherent states at 795 nm (from the VCSEL) and 405 nm (from the pump laser), respectively. The first one reveals only the two successive diffraction orders, while for a half-shorter wavelength, five successive orders are visible. The intensity diffraction orders in *G*^*(1)*^*(****k****)* pattern for classical coherent state at the wavelength 795 nm (close to 810 nm), almost coincide with the even orders of the coherent state at 405 nm. At the same time there is no diffraction lobes of the coherent state at 795 nm in the directions of odd diffraction orders at 405 nm wavelength. The corresponding second-order correlation patterns *G*^*(2)*^(***k***, ***k***′) obtained for these two coherent states are shown in Fig. 2(b,e). In order to represent *G*^*(2)*^*(k*_*x*_, *k*_*y*_, *k*_*x*_′*, k*_*y*_′*)* as a 2D map, we introduce linearized pixel indexes *k* = *k*_*y*_ + *N* *×* *k*_*x*_ (and *k*′ = *k*_*y*_′ + *N* × *k*_*x*_′) that continuously numbers in a line all *N* × *N* pixels within the array, first along the columns in the *k*_*y*_ direction and then changing between the columns in the *k*_*x*_ direction (more details can be found in Supplementary Information). As a clue to interpret such *G*^*(2*)^*(k,k*′) patterns after pixel reshuffling, in Fig. 2(c,f) we provide the corresponding *G*^*(1)*^(*k*) × *G*^*(1)*^(*k*′) patterns for accidental coincidences. To ease the comparison, they are also potted using linearized pixel indexes. For a coherent state one shall have identical patterns^23^ *G*^*(2)*^*(k,k*′*)* = *G*^*(1)*^*(k)* × *G*^*(1)*^*(k*′) while a difference in the *G*^*(2*)^*(k,k*′*)* and *G*^*(1)*^*(k)* × *G*^*(1)*^*(k*′) patterns render visible non classical states. In agreement with these considerations, for both coherent states, the two corresponding patterns are very similar to each other. Even after removal of the accidental coincidences, the diffraction lobes are clearly visible on the main diagonal (directed from the bottom left corner to the top right corner) of the pattern in Fig. 2(b) due to low rate of events^24^ (see Supplementary Information). The off-diagonal lobes are due to detection of photons from two different diffraction orders. The vertical and horizontal lines appear due to possibility to separate variables in the form of *G*^*(1*)^*(k*) *×* *G*^*(1*)^*(k*′*)*. Because the diffraction lobes of 405 nm laser intensity pattern in Fig. 2(d) are just one-pixel wide in the *k*_*y*_-axis direction and there is a strong noisy background due to spurious light, only horizontal and vertical lines are visible in the second-order patterns in Fig. 2(e,f).

In contrasts to this, Fig. 2(g–i) show drastically different *G*^*(1)*^, *G*^*(2*)^ and *G*^*(1*)^ × *G*^*(1)*^ patterns measured in the case of bi-photon state at 810 nm produced in non-collinear SPDC regime^14^. The single-point second-order correlation function *G*^*(2)*^(*k*, *k)* taken along the main diagonal of the second-order pattern in Fig. 2(h) reveals five successive two-photon diffraction orders. The directional angles of the two-photon diffraction lobes coincide with 405 nm diffraction orders of classical photons, thus revealing the effect of quantum diffraction at the de Broglie wavelength of 810 nm/2. In the *G*^*(2)*^*(k*, *k*′*)* map spanned over all pixels of the array, they are represented by five anti-correlation traces directed along the main antidiagonal (from the top-left corner to the bottom-right corner of the pattern), as expected for anti-correlated signal-idler pair. More details on experimental results and modelling of the diffraction of bi-photon state as well as the use of single-point correlation function can be found in Supplementary Information. The *G*^*(1)*^*(k)* × *G*^*(1)*^*(k*′*)* pattern in Fig. 2(i) does not show any signature of anti-correlation traces because the variables *k* and *k*′ are not separable, attesting for the detection of entangled state in each two-photon diffraction order.

## Demonstration of the quantum-classical discrimination by the spatial correlation patterns

**Figure 3:**
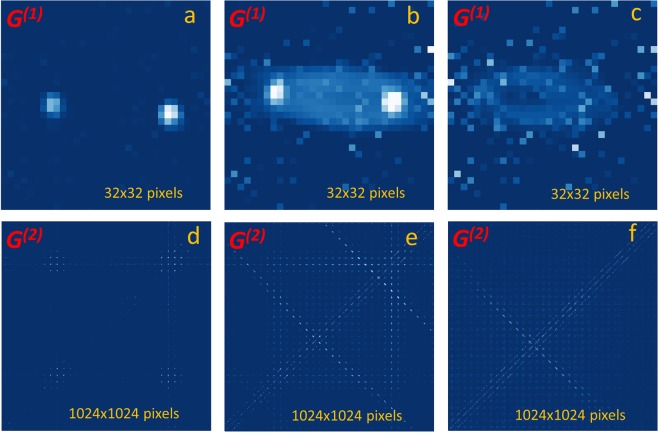
Quantum Classical light Discriminator: Measured first-order G^(1)^(k_x_,k_y_) [(**a–c**)] and second-order G^(2)^(k,k′) correlation patterns [(**d–f**)] in the far field of the echelle grating [(**a,b,d,e**)] and the QCD with a slit after the grating [(**c,f**)]. Left column panels [(a,d)] shows the intensity and joint coincidence patterns for the grating illuminated with a pure coherent state from a laser at 795 nm; Middle column panels [(b,e)] shows these for illumination with the mixed bi-photon and coherent state obtained by combination of SPDC photons and 795 nm laser beams, while in the right column panels [(c,f)], in addition to this a slit is placed in the classically prohibited diffraction order in between the two successive 795 nm diffraction lobes. The second order correlation patterns G^(2)^(k,k′) are plotted after the accidental removal and pixel crosstalk correction and are represented as 2D maps spanned over linearized indexes of the two pixels with SuperEllen array.

Figure 3 demonstrates the QCD operation on a sequence of measurements, showing the first- and second-order diffraction patterns of the echelle grating for the pure coherent state at 795 nm [panels (a) and (d)], for the mixed state of entangled photons at 810 nm and coherent photons at 795 nm wavelength [panels (b) and (e)], and finally the QCD output after the slit for this mixed state [panels (c) and (f)].

Panels (a) and (d) in Fig. 3 display, respectively, *G*^*(1)*^ and *G*^*(2)*^ patterns for the case when only coherent state at 795 nm is incident at the grating and the slit is wide open. They are very similar to the patterns in Fig. 2(a,b) discussed above, showing just two successive diffraction orders at 795 nm. The only difference is that the grating incidence angle is changed for higher angular dispersion. The diffraction lobes at 795 nm are very close to the directional diffraction angels at 810 nm wavelength. The *G*^*(2)*^ pattern in panel (d) shows the two diffraction lobes on the main diagonal due to photons being detected simultaneously in the same diffraction lobe. The two off-diagonal lobes are due to photons detected in different diffraction orders. As discussed above, the weak vertical and horizontal lines are the artifacts caused by the noise events uniformly distributed across the array and detected simultaneously with a photon in one of the diffraction lobes. These features are due to accidental coincidences. The photon state is a classical one because the variables are separable so as the *G*^*(2)*^*(k,k*′) pattern is given by a product of the two intensity patterns *G*^*(1)*^*(k) × G*^*(1)*^*(k*′) (see the discussion to Fig. 2 above).

Panels (b) and (e) in Fig. 3 report on the diffraction of the mixed input state of coherent photons at 795 nm and entangled photons at 810 nm wavelengths produced in non-collinear SPDC regime (the slit is kept wide open). As expected, the two successive diffraction orders at 795 nm wavelength remain visible. The *G*^*(2)*^ pattern now shows additional features due to diffraction of anti-correlated signal-idler pairs. Those are seen as the three anti-correlation traces pointing in the direction of the main antidiagonal. Note that the traces near the main antidiagonal and in the top right quadrant are clearly visible. The third anti-correlation trace in the bottom left quadrant of the figure is barely seen because of the inhomogeneous diffraction pattern of the echelle grating, residue detector pixel crosstalk (seen along the main diagonal) and the noise events detected simultaneously with the photons (seen as vertical and horizontal lines). The principle diffraction lobes of the classical photons at 795 nm wavelength seen on the main diagonal of the *G*^*(2)*^ pattern are located nearby the two anti-correlation traces from the two consecutive even diffraction orders of bi-photons at the de Broglie wavelength of 810 nm/2. The small shift due to the spectral dispersion of the grating has no impact on the generality of the results reported here. Most importantly, the odd *G*^*(2)*^*(k, k)* diffraction order at bi-photon de Broglie wavelength seen as the anti-correlation trace passing near the main antidiagonal of the pattern does not overlap with any principle diffraction lobe of 795 nm light. This can also be appreciated from the *G*^*(1)*^ intensity pattern in Fig. 3(b). The entangled photon state is seen as a blurred cloud, thus attesting that bi-photons are physically present at the diffraction angles prohibited for classical photons of the same optical wavelength (see also Supplementary Information). The *G*^*(2)*^ pattern cannot be any more decomposed as a product of two *G*^*(1)*^ patterns, attesting that the diffracted state has non-classical content and is non-separable.

The effect of the QCD on this input mixed state is pictured in panels (c) and (f) in Fig. 3. The output slit of QCD is now narrowed and positioned halfway between the two principle lobes of the classical diffraction pattern, i.e. to one of the odd diffraction orders of bi-photons at their de Broglie wavelength of 810 nm/2. Thus, only the classically prohibited range of directional angles between the two lobes is passing. The photon pairs passing through the slit can be physically seen in the *G*^*(1)*^ intensity pattern in Fig. 3(c) as a blurred cloud with no signature of classical photons. Respectively, the *G*^*(2)*^ pattern in Fig. 3(f) reveals only one anti-correlation trace if we neglect the residue pixel crosstalk and noise events. The signature of only non-classical correlations of the transmitted photon state testifies that the combination of a diffraction grating and slit discriminates the quantum and classical components. We may thus refer to it as “Quantum-Classical discriminator” (QCD) based on the de Broglie wavelength diffraction.

## Demonstration of the quantum-classical discrimination with temporal correlation patterns

**Figure 4:**
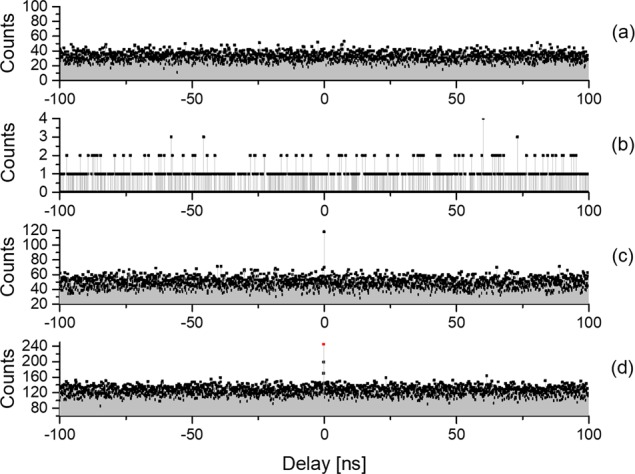
Start-stop histograms of the delayed photon detection events measured at the output of the Quantum-Classical Discriminator illuminated with various input states: (**a**) for input coherent state at 405 nm; (**b**) for coherent state at 795 nm (only residual noise due to spurious light and dark counts are visible); (**c**) for mixed state of a coherent laser at 795 nm and non-collinear SPDC state at 810 nm; (**d**): for mixed coherent 795 nm and collinear SPDC. Data accumulation time: (**a**) 19 h; (**b**) 14 h (**c**): 18 h (**d**): 111 h. The peak to background ratio is 2.5:1 in (**c**) and 2.0:1 in (**d**) after the binning correction of the correlation peak at zero delay (red point).

The spatial correlation patterns *G*^*(2)*^ in Fig. 3 are distorted by the residue pixel crosstalk and noise events, limiting so far the QCD tests to the signal-idler pairs produced in non-collinear SPDC regime. In order to unambiguously confirm that the transmitted light state of the QCD is a non-classical bi-photon state and that QCD operates equally well with collinear signal-idler pairs, we measure its temporal correlations by recording the start-stop histogram of the delayed photon arrivals at the output of QCD, using a beamsplitter (BS), two standalone single-SPAD detector modules and a digital oscilloscope (Fig. 1). Contrary to the spatially resolved measurements, the temporal resolution bin size is 100 ps, which occurs insufficiently large to accommodate for the possible jitter and delay time variations in long-duration measurements, as shown below in the discussion to Fig. 4(d).

Figure 4 shows recorded time-difference histograms for four different input states of QCD operating with the slit selecting the same range of directional angles as in Fig. 3(f).

In panel (a) the input state of QCD contains only coherent photons at 405 nm. This is achieved by introducing a 30 °C temperature offset of the PPKTP crystal from the phase-matching condition for SPDC and temporally suppressing one pass-band filter, to attenuate the pump beam just by 60 dB down to 30 nW level. As the slit is located in the odd diffraction order of 405 nm, each detector reveals counts well above its dark counts. As expected, no photon bunching is observed at zero delay for this coherent state.

Panel (b) displays the case where only the radiation of 795 nm laser is fed at the input of QCD while all output beams are blocked by the slit in the odd diffraction order at the de Broglie wavelength 820 nm/2. The count rate is low, mainly defined by the detector dark counts and spurious light, attesting that no classical photons at 795 nm are present at the output of QCD.

Panel (c) presents the time-difference histogram when the QCD input is probed with the mixed state of coherent photons at 795 nm and non-collinear SPDC bi-photons at 810 nm. We see the appearance of a correlation peak at zero-delay at the output of QCD, having peak-to-background ratio of 2.5:1. This result indicates the presence of pure bi-photon state and thus provides an additional evidence to conclusion drawn from the results in Fig. 3(b) on the QCD operation. Note that the background is defined by accidental events due to dark counts and photons detection from different correlated pairs, while as shown in Fig. 4(b), 795 nm classical photons are suppressed by QCD (see also the Supplementary Information).

So far we have reported results obtained with non-collinear degenerate SPDS photons. In order to prove that observed QCD features are not linked to the angular spread of bi-photons, we repeat the measurement form Fig. 4(c) with the PPKTP crystal temperature increased by a few °C, in the collinear non-degenerate SPDC regime^13,14^. Note that with increasing temperature, the pair production rate lowers^13,14^ and therefore we perform measurements with significantly longer integration time (the integration times are quoted in the figure caption). Panel (d) represents the time-difference histogram for such mixed input state of coherent photons at 795 nm and collinear photon pairs at 810 nm. Once again the coincidence peak at zero delay in Fig. 4(d) indicates presence of bi-photons at QCD output. A careful examination of this histogram reveals the excess correlations in the two consecutive bins near zero delay (two black points above the background), indicating that the bin width was not sufficiently large to fit the measured time difference variations and drift during this long-term measurement. Applying binning to the two excess counts and placing them in the central bin (shown as a red point at zero delay) we find the peak-to-background ratio of 2.0:1. Within the accuracy of the accidental coincidence background due to several photon pairs arriving at detectors in the interval corresponding to their integration time, these experimental results agree with theoretical interpretation given in the Supplementary Information.

## Possible extension of quantum-classical discriminator design

The concept of quantum-classical discrimination with selection of only one order by a single slit may be extended to a mask, containing multiple apertures. Such mask will provide a higher throughput for non-classical photon states and eventually will make possible observation of entangled photon states with order higher than 2. This can be achieved by selecting a set of diffracted beams containing only entangled photon states. We investigate a mask with three slits located at successive odd diffraction orders of the two-photon diffraction pattern of bi-photons at de Broglie wavelength. The mask is placed in the far field of the echelle grating at the location of adjustable slit shown in Fig. 1. Respectively, the SuperEllen SPAD array detector is now used to analyze the far field patterns of the mask.

**Figure 5:**
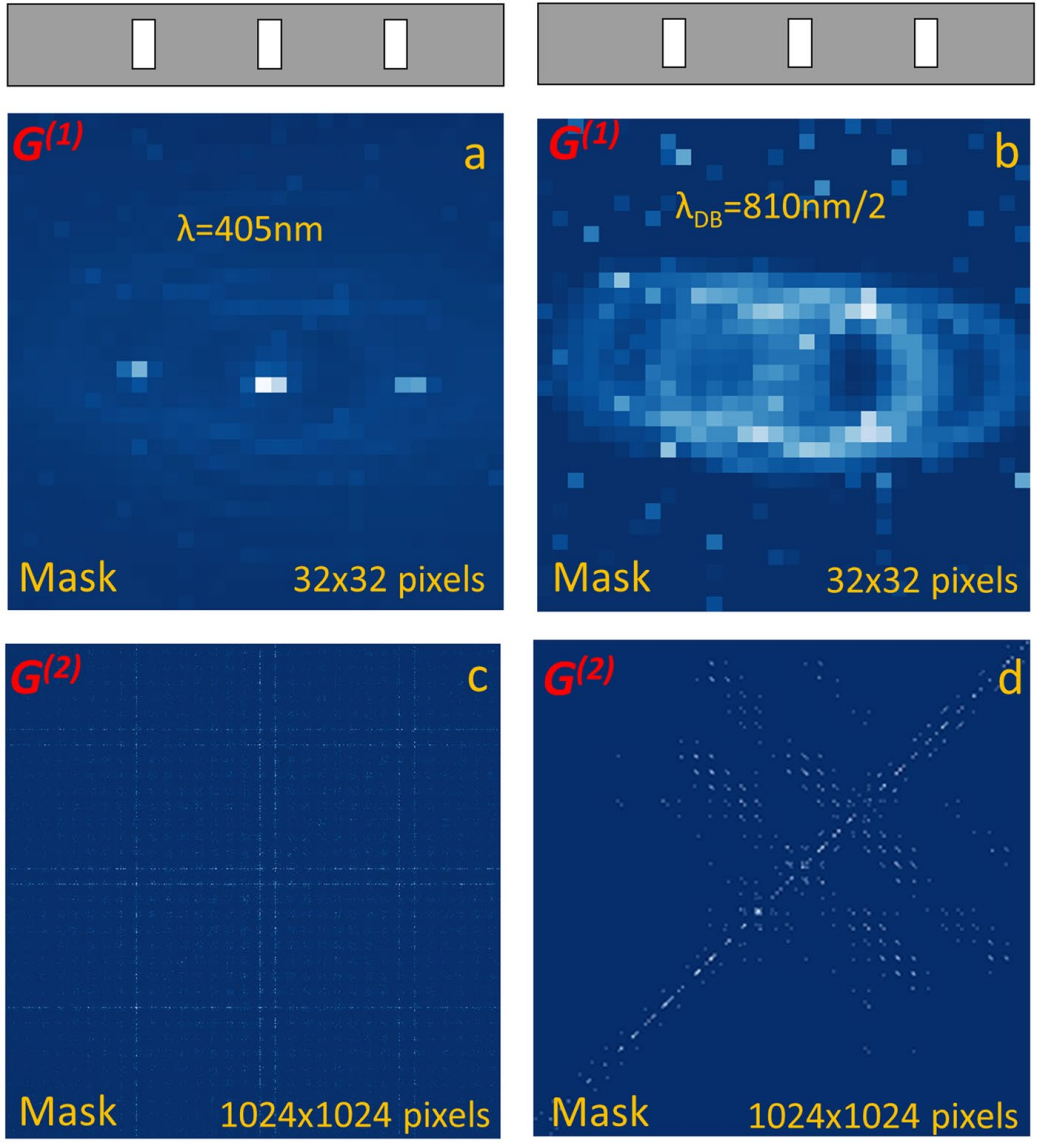
Application of the multiple-slit mask: First-order G^(1)^(k_x_,k_y_) intensity patterns [(**a,b**)] and second-order G^(2)^(k,k′) correlation patterns [(**c,d**)] measured with classical coherent light at 405 nm [(**a,c**)] and with bi-photons produced in non-collinear SPDC regime at 810 nm. The mask slit location with respect to intensity patterns is sketched on the top of G^(1)^ patterns. The second order correlation patterns G^(2)^(k,k′) are plotted after the accidental removal and pixel crosstalk correction and are represented as 2D maps spanned over linearized indexes of the two pixels within the detector array.

The mask is sketched in the top of Fig. 5. It contains apertures corresponding and aligned to the odd orders of 405 nm diffraction pattern. To simplify masks alignment, we temporally suppress one pass-band filter to reduce attenuation of 405 nm beam. This results in 405 nm classical light being of slightly higher power (~30 nW) as compared to SPDC photons power (~6 nW). The *G*^*(1)*^*(k*_*x*_*,k*_*y*_*)* intensity pattern in Fig. 5(a) attests that the slit mask is properly aligned. The presence of weak contribution from SPDC photon pairs is verified by observing the donut-like structures superimposed on laser diffraction pattern. As expected, the measured *G*^*(2)*^*(k,k*′) pattern in Fig. 5(c) reveals only classical component. Within the accuracy to suppressed even diffraction orders, it is similar to the initial pattern for diffraction of 405 nm coherent state in Fig. 2(e).

Figure 5(c,d) show the *G*^*(1)*^*(k*_*x*_*,k*_*y*_*)* intensity pattern and the second order correlation pattern *G*^*(2)*^*(k,k*′) measured after the mask with a pure bi-photon state produced in non-collinear SPDC regime. For this we recover back the 60 dB pass-band filter and reduce the residue 405 nm beam power down to 30 fW. Like in Fig. 2(g), the intensity pattern in Fig. 5(c) shows several donut-like structures superimposed on each other. Like in Fig. 2(h), the multiple anti-correlation traces in *G*^*(2)*^ pattern in Fig. 5(d) are also clearly visible, but surprisingly, the separation between the anti-correlation traces is twice less than in Fig. 2 and the literature case^3,4^ of quantum two-photon diffraction at de Broglie wavelength 810 nm/2, when no mask is applied.

In this measurement, the half-period translation of the mask from the locations of odd diffraction orders at de Broglie wavelength 810 nm/2 to the locations of even orders does not change the *G*^*(2)*^ pattern. The fact that *G*^*(2)*^ fringe period is reduced by a factor of two, provides a hint that a possible theoretical explanation should be linked to a *π*-phase shift between adjacent slits of the mask for classical photons at 810 nm, yielding *2π*-phase shift for bi-photon state, in accordance with the de Broglie wavelength concept. Because the (angular) distance between the slits of the mask is twice larger than the distance between the two-photon diffraction orders of the grating before the slit, the two-photon fringe density in the far field of mask is twice larger as well. In Supplementary Information we provide a simplified theoretical description for such quantum diffraction mask experiment. This mask can be used as an alternative QCD with multiple beam output. Its detailed experimental examination is left for another study.

## Conclusion

We demonstrated a discrimination of entangled states from the classical photons, when photons have the same classical characteristics such as the optical wavelength, propagation direction and polarization. The discrimination is based on the effect of quantum diffraction of the bi-photon state according to its de Broglie wavelength of λ_pair_ = λ/2 instead of the optical wavelength λ of photons composing the pair. It is realized experimentally with the echelle grating and a slit by selecting an odd diffraction order at de Broglie wavelength which is prohibited for classical light.

The predominance of the bi-photons in the output light state after the discriminator is confirmed in two ways, by detection of their spatial and temporal *G*^*(2)*^ correlation patterns. Particularly, for the detection of the spatial correlation patterns, we used a purposely developed SPAD array detector, what itself is also a step forward towards the practical application of entangled photons, e.g. in imaging with resolution beyond the Rayleigh limit.

In the reported validation we used an echelle grating as diffractive element. This element was selected because of its convenience of multiple orders making easy the selection of even and odd orders sequence. In perspective practical applications where entangled photon state purification will be necessary, it may be better to use diffraction gratings with higher efficiency and less orders. On the other side, the echelle grating offers a possibility to select a multiple-beam patterns with purified quantum states. This arrangement can provide a higher throughput but also it may be useful in many other quantum optics setups, replacing complex optical systems with multiple beams splitters.

The reported discrimination effect is based on quantum diffraction of bi-photons, physically present in each two-photon diffraction order at de Broglie wavelength. We may assume that the same effect may be also used for the selection of entangled photon groups of higher orders, using the n-photon diffraction patterns at their corresponding de Broglie wavelength of λ_N_ = λ/N.

## Supplementary information

Supplementary information.
