# Supplementary material for: Discrimination of entangled photon pair from classical photons by de Broglie wavelength
Source: Sci Rep. 2020 Apr 27;10:7087. doi: 10.1038/s41598-020-63833-8 (PMC7184570; doi:10.1038/s41598-020-63833-8)
Supplement: Supplementary file 1 — Supplementary information. [file 41598_2020_63833_MOESM1_ESM.pdf]

SUPPLEMENTARY INFORMATION

Discrimination of entangled photon pair from classical photons by de Broglie wavelength

Valentin Mitev*, Laurent Balet, Nicolas Torcheboeuf, Philippe Renevey and Dmitri L. Boiko

CSEM, rue de l'Observatoire 58, Neuchâtel 2000, Switzerland;

***Contact** author: Valentin Mitev; e-mail: valentin.mitev@csem.ch*

1 SuperEllen functionalities and characterization

The spatial correlation patterns are recorded by a dedicated 32×32 pixels time-resolved SPAD array detector implemented in CMOS and referred herewith as SuperEllen[1,2]. The pixel pitch is of 44.64 µm, the acquisition window is of 30 ns, and resolution of the time-to-digital converter (TDC) is of 160 ps. The coincidence resolution is intentionally put to 480 ps to accommodate for possible TDC delays and jitter across the array The Photon Detection Probability (PDP) is >20 % at 420 nm and >5% at 800 nm. The pixel dark count rate of 470 Hz (on average across the array) is relatively high *vs.* the typical one of ~5-10 Hz for a stand-alone SPAD detector. In addition, the pixel crosstalk effect must be considered in the treatment of the correlation patterns (see below). However, the imager avoids tedious mechanical scanning and allow acquisition of the spatial correlation patterns in a single measurement. The readout time of the imager between frames is 10 µs, which is sufficient for data transfer on a PC and online calculation of the first and second order correlation patterns.

In Figure S1, we illustrate SuperEllen performances (and limitations) in application to the detection of the bi-photons produced via SPDC in a PPKTP crystal[3,4]. The optical setup is essentially the same as shown in Figure 1 in the main paper, except that the echelle grating is replaced by Ag-coated mirror.

Figure S1 displays the Far-Field $G^{(1)}(\mathbf{k})$ and $G^{(2)}(k,k')$ patterns captured by the SuperEllen for non-collinear spectrally-degenerate SPDC, when the crystal temperature is less than the critical temperature[4] ($T<T_c$). In this condition, the wave vectors of signal and idler photons form a cone, i.e. they are anti-correlated in the lateral plane direction. Respectively, the integrated intensity distribution in Figure S1 (a) shows up a peculiar donut-like pattern.

Figure S1 (b) represents the second-order correlation pattern obtained by measuring all pairwise pixel correlations $G^{(2)}(\mathbf{k},\mathbf{k}')$. While $G^{(1)}(\mathbf{k})=G^{(1)}(k_x,k_y)$ pattern can naturally be plotted as a two-dimensional (2D) map in the plane of pixel row k_x and pixel column k_y indexes, the second-order pixel-pairwise correlation function $G^{(2)}(\mathbf{k},\mathbf{k}')=G^{(2)}(k_x,k_y,k_x',k_y')$ is a 4D dimensional distribution spanned over two sets of pixel rows and columns within $N\times N$ array (32×32 in our case). In order to represent it as a 2D map, we introduce a linearized index $k = k_y + N\times k_x$ that continuously numbers in a line all $N\times N$ pixels (1024 in our case) within the array, first along the columns in the k_y direction with increasing row index and then changing between the columns in the k_x direction. The horizontal and vertical axes in Figure S1 (b) indicate two sets of linearized indexes k and k', respectively, enabling to represent all pixel-pairwise correlations $G^{(2)}(k,k')$ in one 1024×1204 pattern. To shorten notation, we use variable k interchangeably to designate either the detector pixel index or wavevector according to the context.

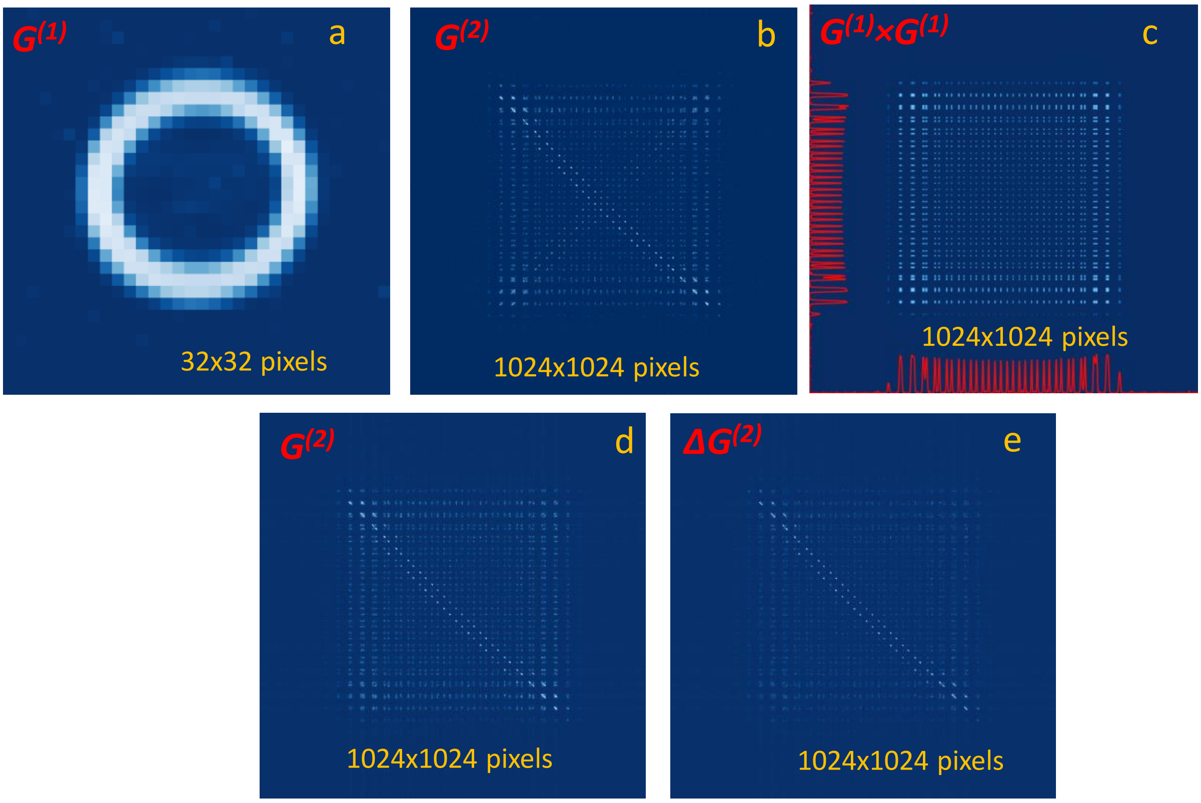

Figure S1. The intensity and correlation patterns of the SPDC beam captured by SuperEllen SPAD array detector. (a) Integrated intensity $G^{(1)}(\mathbf{k})$; (b) measured second order correlations function $G^{(2)}(k,k')$ represented in the coordinates of linearized detector pixel indexes; (c) its classical counterpart $G^{(1)}(k)\times G^{(1)}(k')$ due to accidental coincidences; (d) the same pattern as in (b) but after the pixel crosstalk correction and (e) after the both pixels crosstalk correction and excess correlations due to low count rate removal $\Delta G^{(2)}(k,k')=G^{(2)}(k,k')- G^{(1)}(k)\times G^{(1)}(k')$.

For each pixel pair, the photon arrival times recorded by the sensor are processed to a histogram of time differences between any possible combinations of events including detection of correlated photon pairs but also uncorrelated events due to the dark counts, pixel crosstalk events and spurious light. In function of the count rate and acquisition window width, uncorrelated events produce a triangular pedestal peaked at zero time delay[2,5] of the histogram. In order to extract the correlated events at zero-time delay in excess to the accidental coincidences background, this triangular background is removed from the measured time differences histogram. The excess value at zero delay (within the predefined coincidence window of 480°ps) is used to extract the joint count rate for the pixel pair. In Figure S1 (b), the resulting pixel-pairwise joint count rates $G^{(2)}(k,k')$ in access of accidentals $\propto G^{(1)}(k) \times G^{(1)}(k')$ are plotted across the entire detector array. This difference pattern is expected to render visible non-classically correlated states.

Figure S1 (c) shows a corresponding map of accidental coincidences $G^{(1)}(k) \times G^{(1)}(k')$ as it would be in the case of classical coherent state with the same intensity pattern as in Figure S1 (a). Like the second-order correlation map in panel (b), it is also plotted in function of the linearized indexes of the pixel pair. The red colored curve on the horizontal (vertical) axis indicates how the pixel index linearization procedure transforms the 2D intensity map $G^{(1)}(k_x,k_y)$ from Figure S1 (a) into 1D profile $G^{(1)}(k)$. Although this pixel index linearization procedure resamples the actual intensity profile and makes it hard to appreciate, it is still possible to gain very important insights from the comparison of the patterns in Figure S1 (b) and (c).

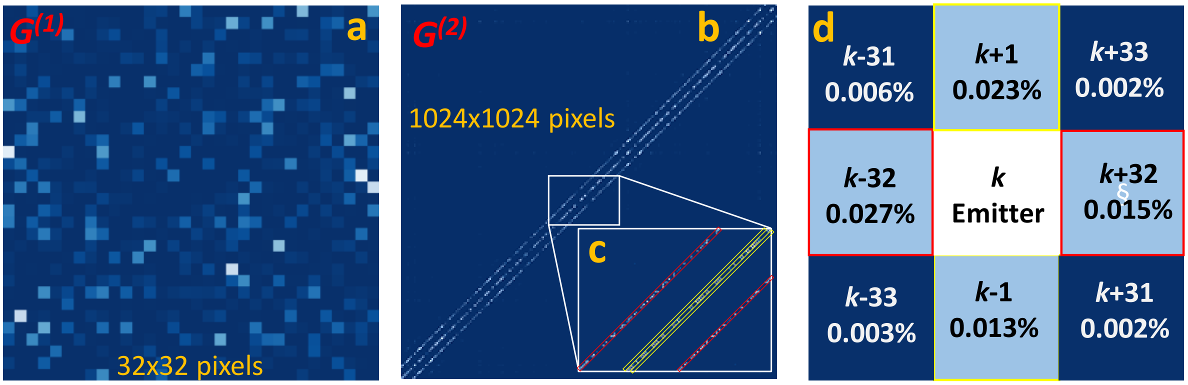

Figure S2. First- and second-order correlation patterns measured with only dark counts. (a) Integrated intensity pattern $G^{(1)}(k_x,k_y)$; (b) joint coincidences pattern $G^{(2)}(k,k')$ plotted as a function of two linearized indexes of the array pixel pair; (c) Zoom of the rectangular area in panel (b); (d) a central pixel with linearized index k (white color) is in a crosstalk with its four nearest neighbors (blue color) and its four next nearest neighbors (dark blue color). The values in the cells indicate pixel indexes and crosstalk probabilities with respect to the central pixel.

In Figure S1 (b), the signal-idler photon anti-correlations are clearly visible along the main antidiagonal (directed from the upper left corner to the bottom right corner). This is due to the fact that the signal and idler photons of a correlated pair hit the detector pixels on the opposite sides of the donut shape in Figure S1 (a), yielding a particular anti-symmetric relationship between the pixel indexes. This anti-correlation feature is not present in the classical correlations pattern $G^{(1)}(k) \times G^{(1)}(k')$ in Figure S1 (c) and hence can be attributed to non-classical correlations. For a classical coherent state one would observe identical patterns[6,7] $G^{(2)}(k,k') = G^{(1)}(k) \times G^{(1)}(k')$ in panels Figure S1 (b) and (c).

Another important difference of the two patterns is linked to the correlations seen on the main diagonal (directed from the bottom left corner to the upper right corner). They are caused by the neighbor pixels crosstalk events and limits partially the capabilities of the SuperEllen detector in sensing of spatially tightly-correlated photon pairs (e.g. in the near-field of PPKTP crystal or in the case of collinear non-degenerate signal-idler pairs).

To illustrate the pixel crosstalk effect, we use pixel dark counts, which normally should not reveal any statistical correlations and should lead to uniform $G^{(2)}(k,k')$ background, due to accidental coincidences. As expected, the measured integrated intensity pattern $G^{(1)}(\mathbf{k})$ shows a random distribution [Figure S2 (a)]. The bright spots indicate pixels with the higher dark count rates. Despite the random location of these pixels, the measured $G^{(2)}(k,k')$ correlation function in Figure S2 (b) reveals a pattern of coincidences along the main diagonal. A zoom shown in Figure S2 (c) allows to deduce that these are the correlations between pixels in nearby columns with (linearized) index difference of ±1 and between nearby rows with pixel index difference of ±32. They are seen due to the nearest- neighbor pixel crosstalk.

In most cases, $G^{(1)}$ patterns do not reveal noticeable distortion from the pixel crosstalk[8]. Thus the bright individual pixels in Figure S2 (a) are not blurred. Nevertheless the measured $G^{(2)}(k,k')$ patterns generally require the crosstalk correction, as seen in Figure S1 (b). This is especially true for low signal count rates, e.g. at the output of QCD. We remove the excess correlations caused by the pixel crosstalk using the considerations and procedures described in[9]. As illustrated in Figure S2 (d), we consider four nearest neighbors and four next nearest neighbor pixels. Their linearized indexes and measured crosstalk probabilities with respect to the central pixel *k* are indicated in the Figure S2 (d). For example, the measured second-order correlations between pixels *k* and *k+1* are corrected for the crosstalk in the following way:

$$G^{(2)}(k,k+1) = \tilde{G}^{(2)}(k,k+1) - \varepsilon_{k,k+1}(G^{(1)}(k) + G^{(1)}(k+1)) \quad \text{(S1)}$$

Here $\tilde{G}^{(2)}(k,k+1)$, $G^{(1)}(k)$ and $G^{(1)}(k+1)$ are the measured second- and first-order correlation functions and $\varepsilon_{k,k+1}$ is the pixel crosstalk probability. Figure S1 (d) illustrates the effect of crosstalk removal on example of $G^{(2)}$ pattern from Figure S1 (b).

After this correction, one would expect to observe only the anti-correlation traces due to non-collinear signal-idler pairs. However, the excess of coherent-state-like correlations above the accidental background is also visible in Figure S1 (d) [compare the square shaped background in Figure S1 (d) and the accidental coincidence pattern in Figure S1 (c)]. We attribute these excess correlations at zero-time delay to low photon number effect, as discussed elsewhere[10]. The excess is inversely proportional to the overall rate of detection events (i.e. due to the signal and idler photons, dark counts, spurious light etc). Therefore, the required correction shall be specifically defined in each particular measurement. In Figure S2 (e), the effect of low count rate is reduced by minimizing the difference $\Delta G^{(2)}(k,k') = G^{(2)}(k,k') - \eta G^{(1)}(k) \times G^{(1)}(k')$ with the coefficient $\eta = \sum_{k,k'} G^{(2)}(k,k') / \sum_k G^{(1)}(k) \sum_{k'} G^{(1)}(k')$. Its validity is attested by improved visibility of the anti-correlation trace in Figure S1 (c) due to non-classically correlated photons. The rational for this procedure stems from the observation that in most of cases, the contribution of non-classical correlations in $\sum_{k,k'} G^{(2)}(k,k')$ is much weaker than the contribution of remaining background $\propto \sum_k G^{(1)}(k) \sum_{k'} G^{(1)}(k')$ due to a low event rate.

In the main paper, we report $G^{(2)}(k,k')$ patterns after accidentals and crosstalk removal, using the data treatment similar to the one applied in Figure S1 (d), without introducing an additional correction for the low rate of events.

2 Correlation width of bi-photons in the near field and far field grating illumination

In the literature on quantum diffraction (or interference) of bi-photons in a Young's double-slit setup[11], one can often spot an intriguing requirement that both photons of a pair shall pass through the same slit in order to reveal quantum diffraction effects. Expectedly, if it is true, it shall be naturally applied to the cases of multiple slit systems, e.g. diffraction gratings. However our analysis below shows that this can only be formalized as a requirement to the correlation width of bi-photons being smaller than the slit width (or grating pitch) in order to observe the two-photon diffraction orders in the measured $G^{(2)}$ pattern[12,13]. At the same time we conclude that the signal and idler photons can be well separated spatially like in the case of non-collinear photon pairs, without decreasing the visibility contrast of the two-photon diffraction pattern $G^{(2)}$. To confirm these considerations, we experimentally study if the positioning of the echelle grating in the far field (FF) or near field (NF) of the SPDC crystal in Figure 1 of the main paper may affect the reported results. We address this point by measuring the bi-photon diffraction patterns $G^{(2)}(k,k')$ for two illumination conditions of the echelle grating pictured in Figure S3, in panel (a) for NF and in panel (b) for FF. Note that in both cases we observe the multiple anti-correlation traces, indicating that the two-photon diffraction occurs equally well under both illumination conditions. (The patterns are shown after the accidental removal and pixel crosstalk correction and are represented using linearized pixel indexes introduced in the previous section of this Supplementary Information.)

The starting point to interpret these results is the beam profile measurements and ray tracing, from which the geometric mean diameter of the pump beam waist in the PPKTP crystal of 12 mm length is about 100 µm. Using the so-called double Gaussian model[14], the estimated correlation width of bi-photons at the output facet of the PPKTP crystal is w_c=23 µm, with the cone angle of the signal-idler pair being of Δk=0.1 µm^{-1} (i.e.,

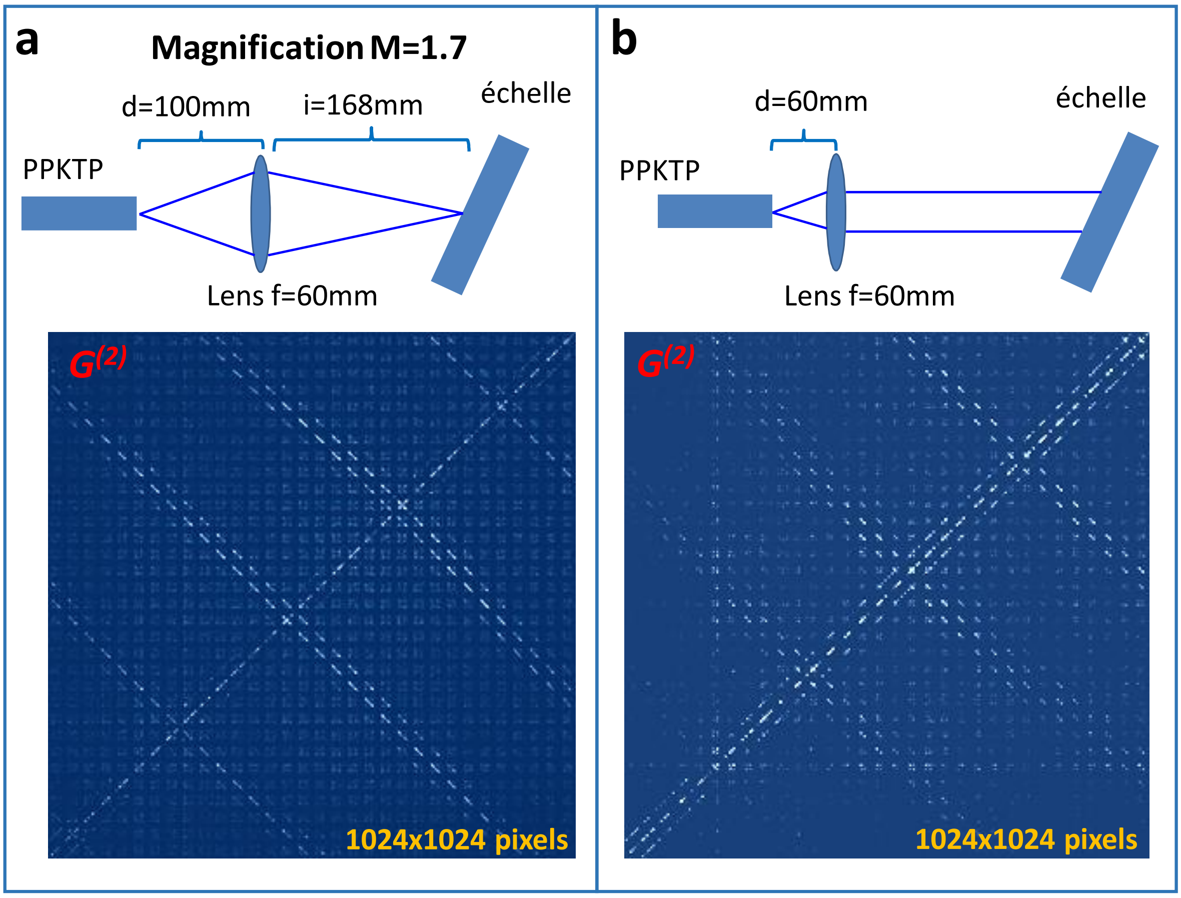

Figure S3. Quantum diffraction of bi-photons under near-field (a) and far-field (b) illumination of the echelle grating from the PPKTP as SPDC source. In each panel the upper part illustrates the setup schematics while the lower part represents the $G^{(2)}(k,k')$ pattern plotted as a function of two linearized indexes of the array pixel pair.

the angular separation of the pair). The anti-correlation width extracted from the width of the donut-like walls, as in Figure S1 (a), is around δk=0.015 μm^{-1}.

In our optical setup, the echelle grating has the groove density of d=31.6 gr/mm. Both for NF and FF illumination experiments, we use an achromatic f=60 mm lens. It should be clarified that alignment of the setup for 810 nm signal and idler photon pair is performed by temporally suppressing the spectral filters and observing the pump beam at 405 nm. For instance, in Figure S3 (a), to achieve NF illumination conditions, the beam waist of the pump within the PPKTP crystal is re-imaged on the echelle grating plane at a magnification of M=1.7. At such magnification, the effective correlation width of bi-photons at the grating plane is of w'_c=39 μm. This is comparable to the echelle period d, and thus the observation of the two-photon interference fringes in $G^{(2)}(k,k')$ is not a surprise. However, in Figure S3 (b) for the FF illumination, the focal spot of the pump beam in the PPKTP crystal is projected almost at infinity at the scale of the setup. This is achieved by focusing it on a screen at 3 m distance. In Figure S3 (b), the signal and idler photons hit the grating at a distance $(\Delta k/k)\cdot f$ =773 μm from each other, thus passing at different grooves of the grating. (This is equivalent to passage of the signal and idler photons at different slits in the equivalent model experiment depicted in Figure S4 and discussed below). According to the criterion quoted from the literature[11], they shall not produce the two-photon quantum interference in the $G^{(2)}(k,k')$ pattern. Nevertheless, the two-photon interference fringes are clearly visible under FF illumination conditions in Figure S3 (b) and appear at nearly

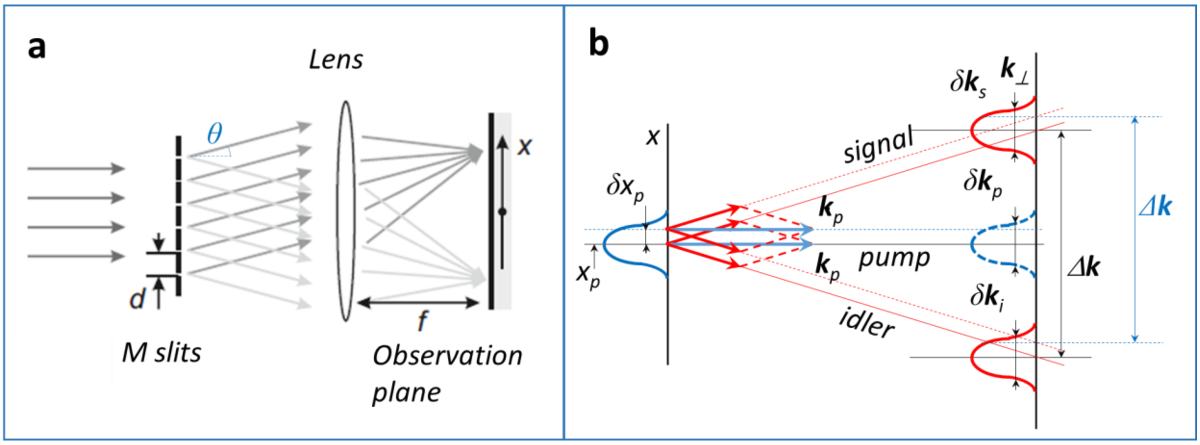

Figure S4. Panel (a): simplified schematics of the quantum diffraction setup. Panel (b): a sketch diagram illustrating near-field and far-field widths of the pump photon beam, bi-photon production and correlations on example of non-collinear signal and idler pair.

same contrast as in the NF illumination case in Figure S3 (a). There is no large difference in the $G^{(2)}$ patterns between the NF and FF illumination cases seen from the experiment.

We find possible explanation by considering the notion of the correlation width of the signal and idler pair in the FF, which is different from their individual wave-vector spreads δk_s and δk_i. This last one estimated from the donut wall width in Figure S1 occurs to be of $(\delta k_{s,i}/k)\cdot f$ = 116 μm in our FF illumination diffraction experiment in Figure S3 (b), which is much larger than the echelle grating pitch d. We thus conclude that the requirement to have the signal and idler photons localized on the same slit (or grating groove) for observing quantum diffraction is not applicable for the FF illumination conditions. It would rather be more suitable to apply quantum path integral technique with the sum (interference) of all possible quantum path amplitudes[15].

For simplicity, as illustrated in the model experiment in Figure S4 (a), we consider a normal incidence on a transmission grating with M slits of the period d. Then we consider a general case of signal and idler photon pair being partially correlated at the grating output:

$$|\Psi\rangle = \sum_{\{s,i\}=1}^{M} c(s,i)\hat{a}_s^{+}\hat{a}_i^{+}|0\rangle, \qquad \text{(S2)}$$

where indexes s and i enumerate grating slits through which signal and idler photons have been transmitted. The summation runs over all possible signal and idler locations in the coordinate space. This are linked to the Cartesian coordinates in the grating plane under the near-filed illumination conditions or transversal wave vector components under the far-field illumination conditions.

In this picture, each slit defines the field mode. Without loss of generality, we assume a Gaussian model of the spatial correlations between the signal and idler photons:

$$c(s,i) = \exp\{-(|s-i|-\Delta)^2/\sigma^2\} \qquad \text{(S3)}$$

Here σ is the correlation width normalized to the grating pitch d; Δ=0 for the NF illumination in Figure S3 (a) and Δ>>σ in the FF case, as in Figure S3 (b). The module sign accounts for the signal-idler wave vector difference being of any sign. For instance, ideally correlated photons under the NF illumination are δ-function like correlated ($\sigma \rightarrow 0$). Such conditions were reproduced in several quantum diffraction experiments where the slits were placed next after the SPDC crystal output facet so as the signal and idler photons can pass through the same slit[11] (σ<<d).

However, under the FF illumination of the grating ($d,\sigma<<\Delta$) the signal and idler photons pass through different slits. We show below that the only condition to observe two-photon interference fringes in the $G^{(2)}$ pattern, is that the uncertainty in the signal and idler relative position $Var(s-i)$ should be smaller than the features to be "measured". In our case, this is the grating pitch size, yielding the same condition $\sigma<d$ as in the case of NF.

Figure S4 (b) illustrates the structure of the state in equations (S2)-(S3) on example of non-collinear signal and idler pair. For instance, the two off-center lobes (red curves and arrows) in the FF of the widths δk_s and δk_i are due to a cross-section profile of the signal-idler distribution that has a donut-like shape in Figure S1 (a). For a collinear pair, the equations (S2)-(S3) do not change but an illustrative figure would be cumbersome because Δ=0 so as the pump, signal and idler occupy nearly same coordinate spaces (x, k).

First, we discuss the NF illumination. The signal and idler operators in equation (S2) commute and hence their coordinates in the NF x_s and x_i, denoted as s and i in equations (S2)-(S3), can be defined simultaneously, meaning that when $\sigma\to 0$, the correlation function $c(s, i)$ approaches a δ-function. Nevertheless, the point in which the pair is created varies across the entire pump beam (shown as blue curves and arrows), yielding a distribution of the width δx_p. It is important to note that the beam size δx_p is not imprinted directly onto the correlation width $\sigma<<\delta x_p$. Therefore, in our simplified model in equations (S2)-(S3), we neglect the final beam size and consider plane waves. We thus consciously avoid the use of so-called double-Gaussian model[16] for which it was concluded that two-photon quantum diffraction in FF illumination is impossible, in contradiction with our experiment in Figure S3 (b).

Considering the FF illumination along the lines of the NF approach, we note that due to the angular width of the pump beam, the axis direction of the total momentum of the signal-idler pair in Figure S4 (b) varies, yielding a distribution δk_p. It contributes directly to the width of the donut walls $\delta k_{s,i}$ as seen in Figure S1 and indicated by red curves in Figure S4 (b). However because the signal and idler operators commute, the transversal wave-vector components of the signal and idler (once again denoted as parameters s and i in equations (S2)-(S3)) can be simultaneously measured in the FF. Their difference Δk (denoted as Δ in equations (S2)-(S3)) is set by the energy and momentum conservation and the phase matching condition along the crystal. Hence the difference $|s-i|-\Delta$ exhibits a δ-function behavior in perfectly correlated signal-idler pair. In reality, it has a finite correlation width σ accounted for in equation (S3). Like the beam size δx_p in the NF, the angular width $\delta k_{s,i}$ of the donut walls in the FF should not be mixed with the correlation width σ of the signal and idler photons. These paraxial beam width parameters δx_p and $\delta k_{s,i}$ are not accounted for by our simple model in equations (S2)-(S3). In contrast to the correlation width σ, the beam width parameters do not bring a fundamental insight on the structure of the $G^{(2)}$ pattern that is inherent to correlated photon pairs. On the other hand, all relevant physical effects related to the correlation width of a pair, are accounted for by our plane wave approximation model in equations (S2)-(S3).

Whether the grating in Figure S4 (a) is placed in the FF or NF of the SPDC crystal, the unitary operator describing the phase accrual after the grating starting from the mth slit (in the mth field mode) to a point in the observation plane at a propagation direction θ, does not change. For both NF illumination and FF illumination cases, it has the following form[17]

$$\hat{u}(m,\varphi)=\exp(-i\hat{a}_m{}^{+}\hat{a}_m m\varphi) \quad \text{(S4)}$$

Here the phase shift between adjacent slits is $\varphi = kd\sin\theta$. We use the phase accrual φ and the observation direction angle θ interchangeably. This is convenient because the diffraction maxima of the classical light states are at the phase angles $\varphi^{(m)}=2m\pi$. Applying transformation from equation (S4) to all modes in equation (S2), we obtain the state in the observation plane expressed in terms of the signal and idler operators at the slits:

$$\left|\Psi_{FF}\right\rangle = \prod_{m}^{M} \hat{u}(m,\varphi)\left|\Psi\right\rangle = \sum_{\{j,k\}=1}^{M} c(j,k)\exp\{-i\varphi(j+k)\}\hat{a}_j^+\hat{a}_k^+\left|0\right\rangle \quad \text{(S5)}$$

For a linear array detector in the observation plan (for simplicity we consider 1D case), each pixel D_ξ registers photons in a specific propagation direction φ_{D_ξ}. We define the corresponding positive frequency part of the field operator by summing up over all modes emitted in this direction[6,7]

$$\hat{E}^{(+)}(\varphi_{D_\xi}) = \int d\varphi \sum_{m=1}^{M} \delta(\varphi_{D_\xi} - \varphi)\hat{a}_m \,. \quad \text{(S6)}$$

We calculate the nth-order correlation functions as the observables for the operators

$$\hat{G}^{(n)}(\varphi_{D_1},\varphi_{D_2},...,\varphi_{D_n}) = \hat{E}^{(-)}(\varphi_{D_n})...\hat{E}^{(-)}(\varphi_{D_1})\hat{E}^{(+)}(\varphi_{D_1})...\hat{E}^{(+)}(\varphi_{D_n}) \quad \text{(S7)}$$

using the wave functions from equation (S5):

$$G^{(n)}(\varphi_{D_1},\varphi_{D_2},...,\varphi_{D_n}) = Tr\{\left|\Psi_{FF}\right\rangle\left\langle\Psi_{FF}\right|\hat{G}^{(n)}(\varphi_{D_1},\varphi_{D_2},...,\varphi_{D_n})\} \,. \quad \text{(S8)}$$

In particular, for the first- and second-order correlation functions we obtain

$$G^{(1)}(\varphi_{D_1}) = 4\sum_{\{m,n,k\}=1}^{M} c(m,k)c(n,k)\exp(-i\varphi_{D_1}(m-n)); \quad G^{(2)}(\varphi_{D_1},\varphi_{D_2}) = 4\left|\sum_{\{m,n\}=1}^{M} c(m,n)\exp(-im\varphi_{D_1} - in\varphi_{D_2})\right|^2 \quad \text{(S9)}$$

First, we examine the quoted literature case[11] of quantum diffraction of bi-photons on a grating in the NF of SPDC crystal (Δ=0) and with the correlation width of the signal and idler photons being much smaller than the grating slit ($\sigma << d$). In Figure S5 we consider the case when σ/d=0.1. Panels (a) and (b) show, respectively, the second-order correlation pattern $G^{(2)}(\varphi_{D1},\varphi_{D2})$ in the observation plane and its counterpart $G^{(1)}(\varphi_{D1})G^{(1)}(\varphi_{D2})$ that would be pertinent to a classical (coherent) photon state. Their difference attests for the non-classical nature of the state shown in panel (a).

The multiple anti-correlation traces in the modelled $G^{(2)}$ pattern reproduce well the main features from the measured pattern in Figure 2 (h) of the main paper. However, there are also some differences that we shall pinpoint and explain. In particular, the modelled $G^{(2)}$ pattern in Figure S5 is uniform in the direction of the main antidiagonal, while the measured one reveals a tiny periodic modulation superimposed on the anti-correlation traces. This difference occurs because in contrast to a 1D linear array detector from our simplistic model, a 2D matrix detector is used in the experiment so as the measured $G^{(2)}$ pattern has a 4D dimensional structure. As discussed in Section 1 of this Supplementary Information, a linearization of the detector pixel indexes is used to represent it as a 2D pattern. It is achieved by numbering the pixels continuously, first along the columns in the direction normal to the diffraction plane and then changing between the columns, in the direction of the diffraction plane. On a large scale, due to slow scanning of the column index, such reshuffling of the pixel pairs from the recorded 4D $G^{(2)}$ pattern into a 2D representation, preserves correlation features in the diffraction plane. However the measured pattern also exhibits additional tiny periodic substructure due to fast repetitive scanning on the pixel row index. This explains why our simplified 1D detector model cannot reproduce all features of the measured $G^{(2)}$ pattern.

Figure S5 (c) shows the cross-sections of the modelled $G^{(2)}$ and $G^{(1)}\times G^{(1)}$ patterns. Note that curve color codding is used to indicate corresponding cross-section locations in the panels (a) and (b). For convenience, we plot them in function of normalized phase angles $\xi = \varphi/2\pi$ such that the integer values $\xi^{(2l)} = \varphi^{(2l)}/2\pi = l$ enumerate the diffraction orders of classical light states [see the green curve in Figure S7 (b)].

Because of nearly δ-correlated signal and idler, the two cross-sections $G^{(2)}(\xi,\xi)$ and $G^{(2)}(\xi+0.5,\xi-0.5)$ along, respectively, the main and shifted diagonals indicated by the dashed red and blue lines in Figure S5 (a) have identical profiles. The first cross-section reveals the so-called single-point correlation function $G^{(2)}$, while for the second function, the two detectors are separated by one order of the classical light diffraction pattern. As it can be anticipated, the period of single-point $G^{(2)}(\xi,\xi)$ function is twice smaller than for the classical light

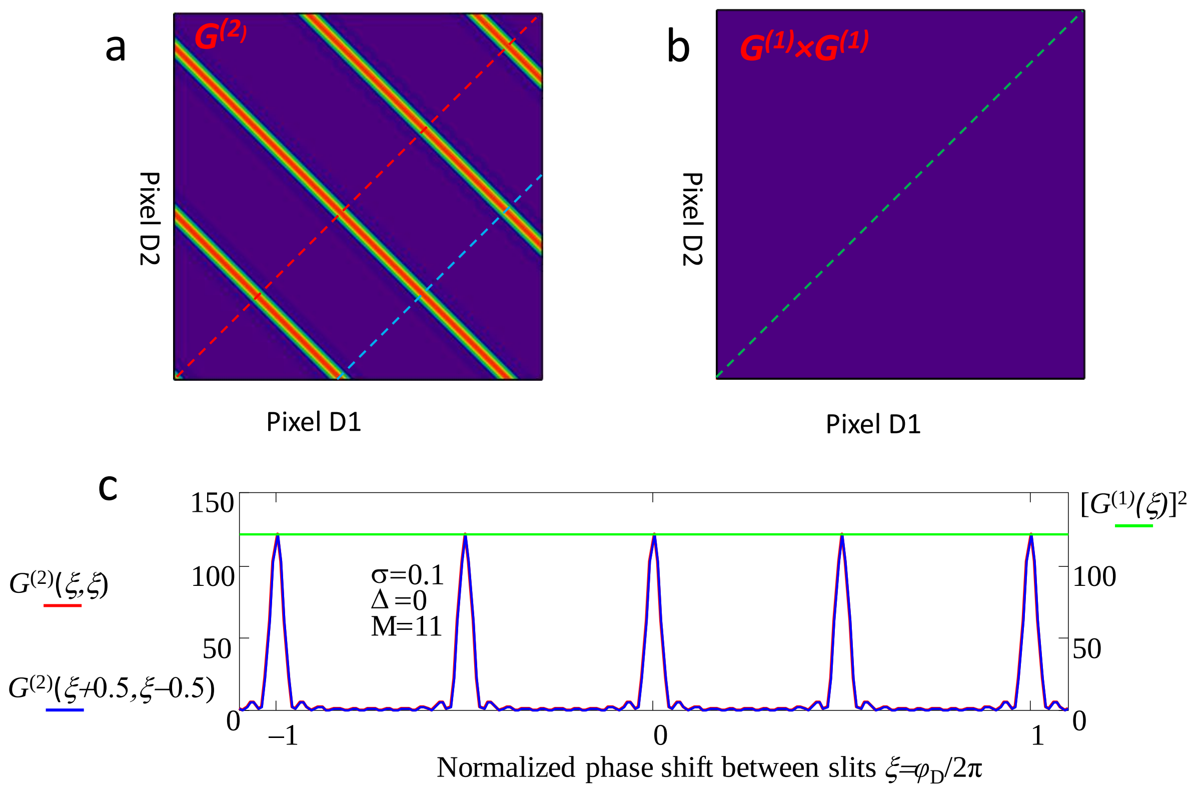

Figure S5. Diffraction of nearly δ-correlated photon pair on a grating placed in the NF of SPDC crystal (Δ=0): Panels (a) and (b) show $G^{(2)}$ and $G^{(1)}\times G^{(1)}$ patterns for all possible combinations of a linear array detector pixels D1 and D2. Curves in panel (c) shows cross-sections along the dashed lines in (a) and (b) of corresponding colors. The coherence width σ of the pair is much smaller than the grating period d (σ/d=0.1). The total number of slits (grooves) is M=11.

state. To avoid possible confusion, note that if, as opposed to moving synchronously both detectors in the case of single-point correlation function $G^{(2)}$, one detector is kept at a fixed location φ_0 (e.g. D1 in the $G^{(2)}(\varphi_{D1},\varphi_{D2})$ pattern in Figure S5 (a)], while the other is moved (e.g. D2 along a vertical line in the pattern), the recorded this way second-order correlation function reveal periodicity of the classical light diffraction pattern.

The intensity distribution pattern $G^{(1)}$ can be appreciated from the product of the first order correlation functions $G^{(1)}\times G^{(1)}$ in Figure S5 (b) as well as from the cross-section profile shown by the green curve in Figure S5 (c). In agreement with Ref.[11], the $G^{(1)}$ pattern exhibits just a constant uniform intensity background. In fact, for δ-correlated signal and idler photons, $m=n=k$ in the equation (S9) for $G^{(1)}$, meaning that both signal and idler photons always pass through the same slit k while all phasors in the sum over different "realizations" are equal to 1, yielding a constant intensity in the observation plane.

All these features can be easily reproduced by a simple analytic model. We consider the state as in equation (S5) assuming δ-correlated photon pairs $c(j,k)=\delta_{j.k}$. This yields from equation (S9):

$$G^{(2)}(\varphi_{D1},\varphi_{D2}) = 4\left|\sum_{n=1}^{M}\exp(-in\varphi_{D1}-in\varphi_{D2})\right|^2 = 4\frac{\sin^2\frac{M(\varphi_{D1}+\varphi_{D2})}{2}}{\sin^2\frac{\varphi_{D1}+\varphi_{D2}}{2}} \qquad \text{(S10)}$$

The two-photon diffraction maxima of the single-point correlation function $G^{(2)}(\varphi_{D1},\varphi_{D1})$ are at the phase angles $\varphi_{D1}=\varphi_{D2}=\varphi^{(m)}=kd\sin\theta^{(m)}=m\pi$, with $\theta^{(m)}$ being the corresponding directional angels. The period is twice smaller than for a classical coherent state, for which m=2l [see equation (S19) below]. The even orders m=2l, which have their classical counterparts, correspond to the integer values $\xi^{(2l)}=\varphi^{(2l)}/2\pi=l$ in Figure S5 (c) while for the odd orders m=2l+1, ξ takes on the half-integer values $\xi^{(21+1)}=l+1/2$ having no classical counterparts.

In Figure S6 we degrade the correlation width, making it comparable to the grating pitch (σ/d=0.8). This results in additional spatial modulation of the periodic $G^{(2)}$ pattern in the anti-diagonal direction. The anti-correlation traces seen in Figure S6 (a) and (c) at a half of the classical light diffraction period attest for the non-classical anti-correlations in the signal-idler pair. The excess of non-classical anti-correlations is clearly seen from the comparison of $G^{(2)}$ pattern in Figure S6 (a) and the one expected for a classical coherent state $G^{(1)}\times G^{(1)}$ shown in Figure S6 (b). However, both the single-point $G^{(2)}(\xi,\xi)$ cross-section along the main diagonal and its one-order shifted counterpart $G^{(2)}(\xi+0.5,\xi-0.5)$ exhibit a period-doubling bifurcation [see the red and blue curves in Figure S6 (c)]. The odd-order lobes due to quantum diffraction are still visible in the two-photon diffraction pattern. However, because the odd-order lobes become of minor amplitude due to reduced coincidence rate, the fundamental spatial frequencies of the two-photon diffraction pattern correspond to the classical light diffraction.

Thus for a finite correlation width σ, the topology of $G^{(2)}$ pattern differs from the ideal delta-correlated pairs in Figure S5. In particular, the odd m=2l+1 and even m=2l two-photon diffraction orders appear differently. Considering directional angle variations near the two-photon diffraction lobes $\varphi=\varphi^{(m)}+\delta\varphi=m\pi+\delta\varphi$ in equation (S5) we can make visible the difference between the odd and even orders:

$$\left|\Psi_{FF}(\varphi^{(m)}+\delta\varphi)\right\rangle=\begin{cases}\sum\limits_{\{j,k\}=1}^{M}c(j,k)\exp\{-i\delta\varphi(j+k)\}\hat{a}_j^{+}\hat{a}_k^{+}\left|0\right\rangle, & m=2l\\ \sum\limits_{\{j,k\}=1}^{M}c(j,k)\exp\{-i\delta\varphi(j+k)\}\exp\{-i\pi(j-k)\}\hat{a}_j^{+}\hat{a}_k^{+}\left|0\right\rangle, & m=2l+1\end{cases} \tag{S11}$$

Thus for the odd diffraction orders m=2l+1, there is an additional π phase shift between adjacent slits. This is a pure quantum effect because for classical light states, a π phase shift between adjacent slits leads to distractive interference of probability amplitudes. For the bi-photon state, constructive interference is possible due to the π phase shift for both signal and idler partita, yielding de Broglie phase shift of 2π for the composite bi-photon state. These quantum diffraction features shall be contrasted with the diffraction of classical light states.

We now make a comparison with the diffraction of coherent light state on the grating. The state just after the grating is a multimode coherent one where the modes are defined by the grating slits[6,7,17].

$$\left|\alpha\right\rangle=\exp(\alpha\sum_{j=1}^{M}\hat{a}_j^{+}-\alpha^{*}\sum_{j=1}^{M}\hat{a}_j)\left|0\right\rangle=\prod_{j=1}^{M}\left|\alpha^{(j)}\right\rangle \tag{S12}$$

The same state in the FF of the grating, i.e. in the observation plane of Figure S4 (a), is obtained by the unitary transform from the equation (S4) at each slit, yielding

$$\left|\alpha_{FF}\right\rangle=\prod_{m}^{M}\hat{u}(m,\varphi)\left|\alpha\right\rangle=\prod_{j=1}^{M}\left|\alpha^{(j)}\exp\{-i\varphi j\}\right\rangle. \tag{S13}$$

The n-th order correlation function reads[6]

$$\begin{aligned}&G^{(n)}_{coh.}(\varphi_{D1},\varphi_{D2},...,\varphi_{Dn})=Tr\{\left|\alpha_{FF}\right\rangle\left\langle\alpha_{FF}\right|\hat{G}^{(n)}(\varphi_{D1},\varphi_{D2},...,\varphi_{Dn})\}\\&=\left|\alpha\right|^{2n}\left|\sum_{j=1}^{M}\exp\{-i\varphi_{D1}j\}\right|^{2}\left|\sum_{k=1}^{M}\exp\{-i\varphi_{D2}k\}\right|^{2}...\left|\sum_{k=1}^{M}\exp\{-i\varphi_{Dn}m\}\right|^{2}=G^{(1)}(\varphi_{D1})G^{(1)}(\varphi_{D2})...G^{(1)}(\varphi_{Dn})\end{aligned} \tag{S14}$$

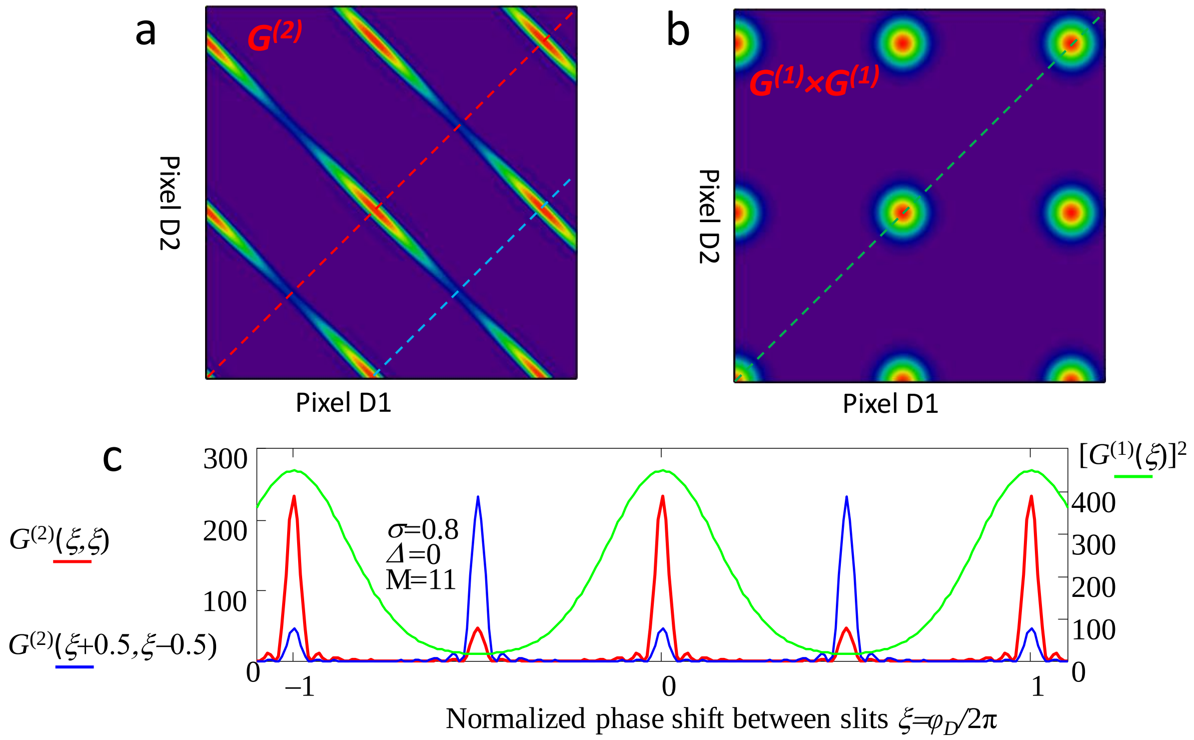

Figure S6. Diffraction of partially correlated photon pair on a grating placed in the NF of SPDC crystal (Δ=0): Panels (a) and (b) show $G^{(2)}$ and $G^{(1)}\times G^{(1)}$ patterns for all possible combinations of a linear array detector pixels D1 and D2. Curves in panel (c) shows cross-sections along the dashed lines in (a) and (b) of corresponding colors. The coherence width σ of the pair is comparable to the grating pitch d (σ/d=0.8). The total number of slits (grooves) is M=11.

In particular, for the first- and second-order correlations we get the following expressions:

$$G^{(1)}_{coh.}(\varphi_{D1}) = |\alpha|^2 \left| \frac{\sin \frac{M\varphi_{D1}}{2}}{\sin \frac{\varphi_{D1}}{2}} \right|^2, \quad G^{(n)}_{coh.}(\varphi_{D1},\varphi_{D2}) = |\alpha|^4 \left| \frac{\sin \frac{M\varphi_{D1}}{2}}{\sin \frac{\varphi_{D1}}{2}} \right|^2 \left| \frac{\sin \frac{M\varphi_{D2}}{2}}{\sin \frac{\varphi_{D2}}{2}} \right|^2 \qquad \text{(S15)}$$

The diffraction maxima of the correlation patterns are at the phase angles $\varphi^{(2l)} = kd\sin\theta^{(2l)} = 2l\pi$ with $\theta^{(2l)}$ being the corresponding directional angels and $\delta\varphi = 4\pi / M$ being the width of the lobes defined by the first zeros of the count rate. The period of single-point correlation function $G^{(2)}$ is twice larger than for an ideal δ-correlated bi-photon state, as it may be seen from the equation (S10). The last effect is therefore said to provide a resolution improvement. In this paper we use the fact that an additional QCD slit placed in the observation plane at an odd order $\varphi^{(2l+1)} = (2l+1)\pi$ will block all classical photons.

This shall be contrasted with the diffraction of a bi-photon state. The first order correlation function $G^{(1)}$ for the ideal bi-photon state in Figure S5 reveals uniform distribution of the count rate in the observation plane, as seen in Figure S5 (b). It follows that both the count rate $G^{(1)}$ and joint coincidence rate $G^{(2)}$ do not vanish in the direction of the QCD slit $\varphi^{(2l+1)} = (2l+1)\pi$.

It is interesting to note, that in Figure S6 (b) obtained for the finite correlation width σ of the signal and idler pair, $G^{(1)}$ shows diffraction maxima in the directions corresponding to classical light diffraction. From the structure of the phasors in the $G^{(1)}$ correlation function in equation (S9), this shall be interpreted as if the signal and idler can pass with non-zero probability through a pair of adjacent slits in order to produce intensity

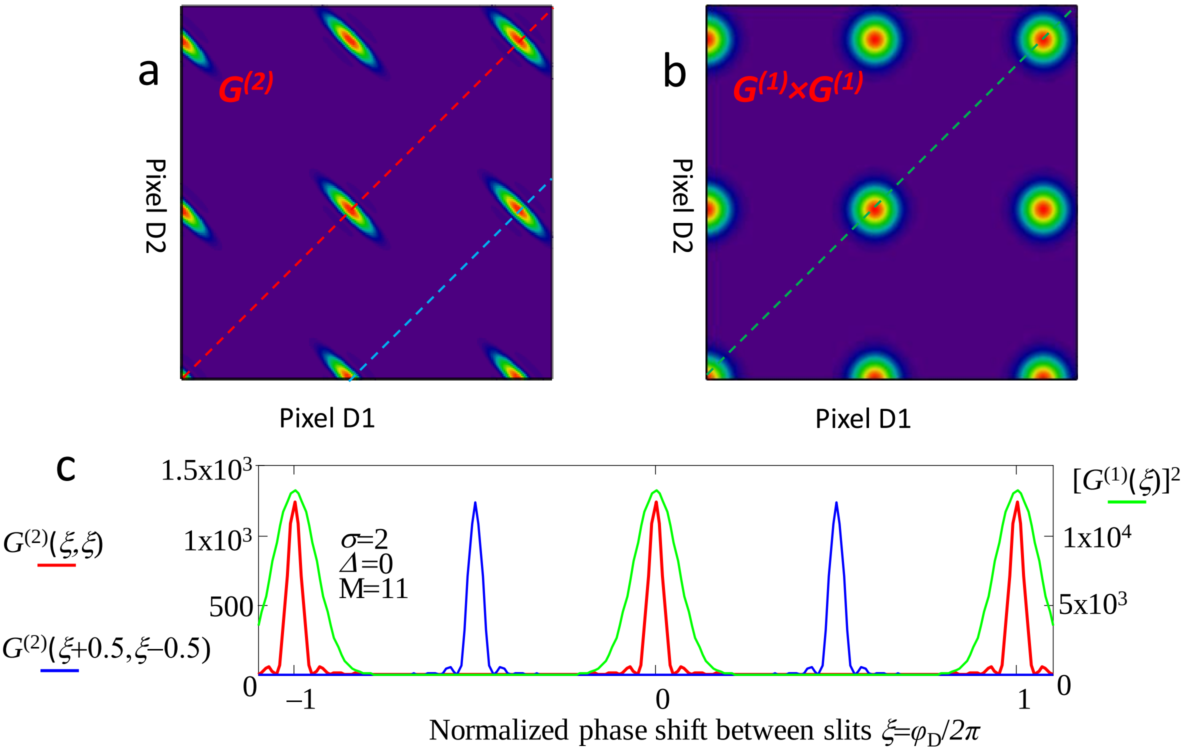

Figure S7. Diffraction of weakly correlated photon pair on a grating placed in the NF of SPDC crystal (Δ=0): Panels (a) and (b) show $G^{(2)}$ and $G^{(1)}\times G^{(1)}$ patterns for all possible combinations of a linear array detector pixels D1 and D2. Curves in panel (c) shows cross-sections along the dashed lines in (a) and (b) of corresponding colors. The coherence width σ of the pair is exceeds the grating pitch d (σ/d=2). The total number of slits (grooves) is M=11

interference fringes in the observation plane. Importantly, the probability to detect both photons of the pair in one of the odd orders $\varphi^{(2l+1)}=(2l+1)\pi$ does not vanish. Moreover, the normalized single-point second-order correlation function $G^{(2)}(\varphi_{D1},\varphi_{D1})/[G^{(1)}(\varphi_{D1})]^2$ is greater than 1 in the direction of the odd diffraction orders (it is of 2.5 in condition of Figure S5), indicating that signal and idler photons tend to pass in a pair through the QCD slit when it is placed in the observation plane as in the setup shown in Fig. 1 of the main paper.

Figure S7 shows another example for the finite correlation width, but this time, for the width being larger than the grating pitch. In this particular example we use $\sigma=2d$. At first sight, a quite different behavior is obtained when $\sigma>d$. It is usually believed that no resolution improvement is possible when correlation width is larger than the features to be resolved. Indeed, only the even-order two-photon diffraction maxima are now clearly visible in the $G^{(2)}$ pattern in Figure S7 (a). They are located at the classical light diffraction lobes in the $G^{(1)}\times G^{(1)}$ pattern [Figure S7 (b)]. There is no signature of the odd two-photon diffraction orders. Therefore, a slit installed in the observation plane at an odd two-photon diffraction order $\varphi^{(2l+1)}=(2l+1)\pi$ will not behave as a QCD.

Nevertheless there are features in Figure S7 (a) that clearly distinguish it from the classical state. Thus, instead of being symmetric on coordinates of two detectors like the $G^{(1)}(\varphi_{D1})\times G^{(1)}(\varphi_{D2})$ pattern in Figure S7 (b), the diffraction lobes in $G^{(2)}(\varphi_{D1},\varphi_{D2})$ pattern are elongated in the anti-diagonal direction, thus partly reproducing non-classical anti-correlations from the patterns in Figure S5 (a) and Figure S6 (a). A quick examination of the $G^{(2)}$ function in equation (S9) shows that the finite correlation width of the signal-idler pair $\sigma\neq0$ results in

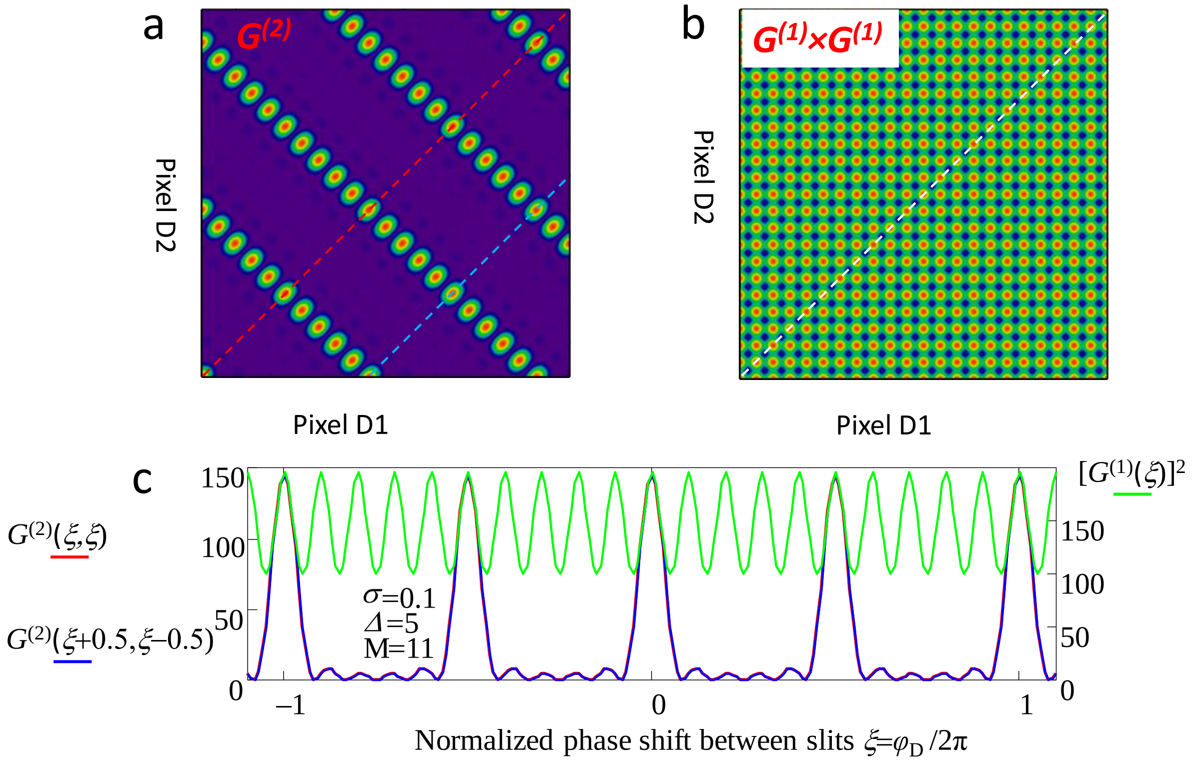

Figure S8. Diffraction of nearly δ-correlated non-collinear photon pair on a grating placed in the FF of SPDC crystal (Δ/σ>>1): Panels (a) and (b) show $G^{(2)}$ and $G^{(1)}\times G^{(1)}$ patterns for all possible combinations of a linear array detector pixels D1 and D2. Curves in panel (c) shows cross-sections along the dashed lines in (a) and (b) of corresponding colors. The coherence width σ of the pair is much smaller than the grating period d (σ/d=0.1) while signal-idler separation is Δ/d=5 grating periods. The total number of slits (grooves) is M=11.

modulation of the initial two-photon diffraction pattern from the equation (S10) in the two orthogonal directions along the main diagonal and antidiagonal:

$$G^{(2)}(\varphi_{D1},\varphi_{D2}) \propto \frac{\sin^2 \frac{M(\varphi_{D1}+\varphi_{D2})}{2}}{\sin^2 \frac{\varphi_{D1}+\varphi_{D2}}{2}} \left(1+\sum_{k=1}^{\infty} c_k \cos\frac{k(\varphi_{D1}+\varphi_{D2})}{2} \cos\frac{k(\varphi_{D1}-\varphi_{D2})}{2}\right)^2 . \qquad \text{(S16)}$$

The location of two-photon diffraction lobes at $\varphi_{D1}+\varphi_{D2}=2m\pi$ in the first term does not change as well as the width of the lobes in the direction of the main diagonal ($\varphi_{D1}=\varphi_{D2}$). For example, for the single-point cross-section $G^{(2)}(\varphi_{D1},\varphi_{D1})$ along the red dashed line in Figure S7 (a), the width of the two-photon diffraction lobes defined by the first zeros of the coincidence count rate is $\delta\varphi_D = 2\pi/M$, i.e. twice smaller than for the classical light diffraction pattern in equation (S15). This quantum narrowing is independent on the ratio between the correlation width of the signal-idler pair σ and the width of the slits (or grooves) *d*.

Finally, at the end of this section we examine the case when diffraction grating is placed in the FF of the SPDC crystal. Assuming that the signal-idler cone [Figure S4 (b)] is collimated at infinity, the signal and idler photons from a pair hit the grating at a fixed distance Δ≠0. Figure S8 shows the results of calculations for a small correlation width σ<<d, as in Figure S5, but this time for Δ≠0. The $G^{(2)}$ pattern in Figure S8 (a) strongly resembles the one for NF illumination in Figure S5 (a). For instance, it also reveals a periodic set of anti-correlation traces at both even and odd two-photon diffraction orders. However, likewise the intensity

distribution $G^{(1)}$, these anti-correlations are now modulated due to interference caused by a phase difference of the signal and idler photons passing at different slits, revealing the spatial quantum beating[19].

It follows that even though the signal and idler photons are not localized to a domain smaller than the features of the object, quantum diffraction can be observed. This is confirmed in experiment illustrated in Figure S3 (b), in which maximum separation of the signal and idler photons can reach Δ/d=19 periods of the grating. However two-photon diffraction pattern in Figure S3 (b) do not reveal longwise modulation of the two-photon fringes shown in Figure S8. This can be attributed to several reasons. Firstly, the 2D matrix detector is used in experiment so as the detector pixels in the joint coincidence rate pattern shown in Figure S3 (b) have been reshuffled to allow 2D representation (see Section 1 and discussion to Figure S5 in this Supplementary Information). Secondly, the signal and idler photons form a cone of possible *k* vector states (Figure S1), being separated with variable number of grating slits. Respectively they produce modulation of two-photon diffraction pattern $G^{(2)}$ at different spatial frequencies. After summation over different signal and idler states, the resulting modulation is washed out. Finally in our setup, the 32×32 pixel detector has insufficient spatial resolution to resolve modulation fringes at large separation of the signal and idler photons.

3 Periodic slit mask effect on the two-photon diffraction pattern

In order to get insight about the measured $G^{(2)}$ patterns in the FF of the grating with the slit mask shown in Figure 5 of the main paper, we utilize diffracted bi-photon state representation from the equation (S11) showing the difference between the odd $m=2l+1$ and even $m=2l$ diffraction orders explicitly. We procced in two steps. To begin with, we consider a mask with *N* slits positioned in the FF of the grating at even orders $m=2l$, as shown in Figure S9. This corresponds to the top row in equation (S11). In order to simplify our analysis, we assume delta-correlations between collinear signal and idler photons [σ→0 and Δ=0 in equation (S3)]. Summing up over all even orders $m=2l$ and over all slits of the mask, we obtain the output state of the mask. On the second step, as indicated Figure S9, we let it propagate and analyze it in the FF of the mask. Now this output state of the mask replaces the initial state equation (S2). Considering after that diffraction on the mask along the lines of the previous analysis in equations (S2)-(S9), we obtain a state in the observation plane labeled as FF of the mask in Figure S9:

$$\left|\Psi_{mask}\right\rangle = \sum_{l=1}^{N/2}\left|\Psi_{FF}^{(m=2l)}\right\rangle \propto \sum_{k=1;l=1}^{M,N/2}\left(\hat{a}_{k,2l}^{+}\right)^{2}\left|0\right\rangle \propto \sum_{l=1}^{N/2}\left(\hat{a}_{2l}^{+}\right)^{2}\left|0\right\rangle . \qquad \text{(S17)}$$

In equation (S17), a pair of indexes {*k*,2*l*} enumerates and distinguishes modes according to their quantum paths[15] through the grating slit with index *k* and the mask slit with index 2*l*. Using this state to calculate the correlation functions in equation (S8), one notices that the sums over the indexes *k* are always complete, while there is no phasor terms associated with *k*. Therefore, to shorten notation, this index can be also omitted in equation (S17). The resulting intensity diffraction pattern $G^{(1)}$ and two-photon diffraction pattern $G^{(2)}$ can be easily obtained along the lines of equation (S10). Accounting, as depicted in the schematic illustration in Figure S9, that the effective angular distance between the slits of the mask is twice larger $\varphi_{mask} = 2kd\sin\theta_{mask}$ (as opposed to $\varphi = 2kd\sin\theta$ after the grating), we obtain a similar expression for the two detectors located in the FF of the mask:

$$G_{mask1}^{(2)}(\varphi_{D1.mask},\varphi_{D2,mask}) = 4\left|\sum_{n=1}^{M}\exp(-in\varphi_{D1,mask} - in\varphi_{D2,mask})\right|^{2} = 4\frac{\sin^{2}\frac{M(\varphi_{D1,mask}+\varphi_{D2,mask})}{2}}{\sin^{2}\frac{\varphi_{D1,mask}+\varphi_{D2,mask}}{2}} \qquad \text{(S18)}$$

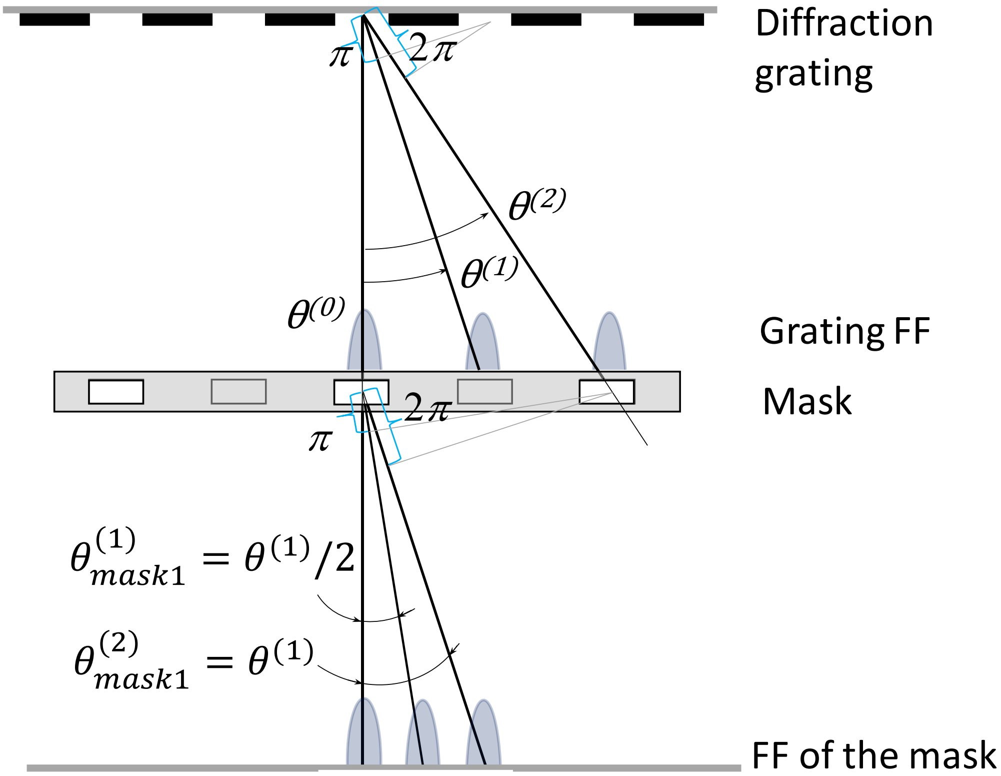

Figure S9. Schematics of bi-photon diffraction on the echelle grating followed by the mask. Note π phase shift between adjacent wavelets of each signal and idler to a diffraction maximum (as opposed to 2π shift for classical photons).

We remind that quantum effects of diffraction at de Broglie wavelength are best observed when two detectors are moved synchronously, e.g. considering a single-point correlations (see the discussion to Figure S5). The two-photon diffraction maxima of the single-point correlation function $G^{(2)}_{mask}(\varphi_{mask}, \varphi_{mask})$ are now twice denser, at the directional angles $\theta^{(q)}_{mask}$ defined by $\varphi^{(q)}_{mask} = 2kd\sin\theta^{(q)}_{mask} = q\pi$. These shall be compared with directional angles $kd\sin\theta^{(m)} = m\pi$ in equation (S10) for the initial unmasked pattern, which contains thus only the even orders of the two-photon diffraction pattern after the mask $\sin\theta^{(m)} = \sin\theta^{(2m)}_{mask}$.

Figure S9 provides a sketch for an easy-to-grasp interpretation. First, we consider quantum diffraction at the grating. A phasor between the adjacent slits to the first two-photon diffraction maximum $\theta^{(1)}$ is π, as indicated in the figure (the zero-order maximum $\theta^{(0)}$ is on the vertical line). For bi-photon state, this yields *2π*-phase shift when both photons of the pair pass through the adjacent slit and are both detected in $\theta^{(1)}$ direction [see also equation (S4)]. The *2π*-phase shift difference for the quantum paths of the pair between adjacent slits of the grating yields constructive interference of the two-photon probability amplitudes from different slits of the grating, in accordance with the de Broglie wavelength concept. For comparison, in the case of classical light diffraction, the first constrictive interference would occur only for the phasor of *2π* between adjacent slits, in the direction designated as $\theta^{(2)}$ in Figure S9. Thus the wavefunction amplitudes of bi-photons at the two adjacent open slits of the mask (at $\theta^{(0)}$ and $\theta^{(2)}$) are in-phase.

Considering along the same lines the two-photon diffraction at the mask, the first diffraction maximum in the single-point correlation function after the mask $G_{mask}^{(2)}$ occurs in the direction $\theta_{mask}^{(1)}$ corresponding to the phasor *π* between adjacent open slits of the mask. The signal and idler photons may both pass through the same open slit or may pass through different slits. In the case when they both pass through the slit at $\theta^{(2)}$ in the direction of $\theta_{mask}^{(1)}$, this add additional *2π*-phase shift for the quantum path of bi-photon, interfering thus constructively with the bi-photon probability amplitude passing through the slit at$\theta^{(0)}$. At the same time, when signal and idler partitas pass different slits, the two amplitudes interfere destructively with each other and cancel out each other. As a result, the constructive interference of the bi-photon probability amplitudes is held in the direction $\theta_{mask}^{(1)}$. Because the (angular) distance between the slits of the mask is twice larger than the distance between the two-photon diffraction orders of the grating, the two-photon fringe density in the FF of mask is twice larger as well.

In a similar way we may prove the appearance of the half-period two-photon fringes for the mask placed at the odd two-photon diffraction orders of the grating *m*=2*l*+1 [see the second row in equation (S11)]. Thus in both locations, the mask produces identical two-photon diffraction patterns $G_{mask}^{(2)}$. When the mask is placed in odd two-photon diffraction orders of the grating, which are prohibited for classical light of the same wavelength, it can be used as a quantum -classical discriminator.

4 Temporal correlations

It is well known that a bi-photon state entering the two input ports of a beam splitter (BS) produces Hong-Ou-Mandel (HOM) dip[20] in the delayed joint coincidence detection at the two BS output ports. Here we consider a different case from our experimental setup, when both signal and idler photons are fed to the same input port of BS and are defined by a wavefunction

$$\left|\Psi_{1p}\right\rangle = \sum_{\{j,k\}} c(j,k)\hat{a}_j^+\hat{a}_k^+\left|0\right\rangle, \tag{S19}$$

where $\hat{a}_k^+$ is the photon creation operator at the input port *a* and in the temporal mode *k*. Thus both photons of the pair in equation (S19) enter the same port of BS. Note that this model does not account for possibility of accidental coincidences of photons from different pairs.

In contrast to this, for a two-port input state in HOM experiment, one dagger operator per input port shall be used, yielding

$$\left|\Psi_{2p}\right\rangle = \sum_{\{j,k\}} c(j,k)\hat{a}_j^+\hat{b}_k^+\left|0\right\rangle. \tag{S20}$$

Along the lines of equation (S3), we consider a Gaussian correlation function $c(i_1,i_2)$ to account for the temporal correlation between the signal and idler photons

$$c(i_1,i_2) = c(i_2,i_1) = \exp\{-(i_1-i_2)^2/\sigma^2\}, \tag{S21}$$

where *σ* is the correlation time and the indexes i_1 and i_2 refer to the temporal modes.

Next we account for the phase shift and time delay between the ports of the BS, using the unitary operators[17]

$$\hat{u}(m,\delta) = \exp(-i\hat{a}_{m+\delta}{}^+\hat{a}_m\psi\delta) \tag{S22}$$

Here $\psi = \omega\cdot 1$ is the phase accrual per unit of time (we denote it as “1”) and *δ* is the delay in mode *m* between BS output and input. The delay $\psi\delta$ can be varied by moving the BS and/or detectors. For instance, for an input Fock state in the temporal mode *i*, we find a delayed state:

$$\prod_m \hat{u}(m,\delta)\left|n_i\right\rangle = \exp(-i\hat{a}_{i+\delta}^{+}\hat{a}_i\psi\delta)\left|n_i\right\rangle = \exp(-in\psi\delta)\left|n_{i+\delta}\right\rangle \quad \text{(S23)}$$

Following the BS analysis from Ref.[20] and explicitly introducing the delay from equation (S22), we find the positive-frequency field operators at the output ports of the BS

$$\begin{bmatrix} E_1^{(+)}(i) \\ E_2^{(+)}(k) \end{bmatrix}_{BS} = \begin{bmatrix} \sqrt{T}\hat{a}_i + i\sqrt{R}\hat{b}_{i+\delta}e^{-i\psi\delta} \\ i\sqrt{R}\hat{a}_{k-\delta}e^{i\psi\delta} + \sqrt{T}\hat{b}_k \end{bmatrix} \quad \text{(S24)}$$

For single-port bi-photon input state from equation (S19), we obtain the following two-time correlation functions $G^{(2)}$ at the BS output:

$$\begin{aligned} G_{12}^{(2)}(i-k,\delta) &= Tr\{\left|\Psi_{1p}\right\rangle\left\langle\Psi_{1p}\right|\hat{E}_1^{(-)}(i)\hat{E}_2^{(-)}(k)\hat{E}_2^{(+)}(k)\hat{E}_1^{(+)}(i)\} = 2TR\left|c(k-\delta,i)\right|^2 \\ G_{11}^{(2)}(i-k,\delta) &= Tr\{\left|\Psi_{1p}\right\rangle\left\langle\Psi_{1p}\right|\hat{E}_1^{(-)}(i)\hat{E}_1^{(-)}(k)\hat{E}_1^{(+)}(k)\hat{E}_1^{(+)}(i)\} = 2T^2\left|c(k,i)\right|^2 \\ G_{22}^{(2)}(i-k,\delta) &= 2R^2\left|c(k-\delta,i-\delta)\right|^2 \end{aligned} \quad \text{(S25)}$$

For detectors with the response times shorter than the correlation time of the pair σ, these expressions provide a good approximation to experimentally measured histograms of the difference of arrival times $\xi=i-k$ of the signal and idler photons. Otherwise, in the case of slow detectors, one has to account for the detector integration time (below denoted as M) by summing equation (S25) over M consecutive indexes i and M consecutive indexes k in function of the average time difference of detection events $\xi=\langle i-k\rangle$. Note that using the model from equation (S19), we assume that only one photon pair arrives at detectors during interval M and no accidental coincidences is possible due to detection of photons from different pairs.

In contrast to HOM dip in the output cross-correlation function for two-port input state, all self- and cross-correlation functions in equation (S25) for the single port input state reveal a coincidence peak of the width defined by the detector response time M, independently of whether the arm delay time δ or the correlation difference time $\xi=\langle i-k\rangle$ is varied [see Figure S10 (a) and (b)]. As discussed above, this provide a true picture at very low rate of photon pairs. In realistic experiment, several photon pair may arrive at detectors within the integration time interval. Such accidental coincidences will shift the curves up in Figure S10 (a) and (b) by adding a constant background.

For an ideal 50:50 BS in Figure S10 (a) and (b), the joint coincidence rates for self-correlations $G_{11}^{(2)}(0,0)$ and cross-correlations $G_{12}^{(2)}(0,0)$ at zero time difference ξ and at zero arm delay δ are the same, in agreement with the structure of an ultimate output state in the case of delta-correlated photons (σ→0)

$$\left|2,0\right\rangle_{out} = T\left|2,0\right\rangle - R\left|0,2\right\rangle + i\sqrt{2TR}\left|1,1\right\rangle \quad \text{(S26)}$$

These results allow one to attribute the correlation peak in the experimental data from Figure 4 (c) and (d) of the main paper to observation of bi-photon state.

This behavior is drastically different from the HOM experiment when the signal and idler photons enters BS at two different input ports. The corresponding delayed coincidence rates for the self- and cross-correlations with slow detectors are plotted in Figure S10 (c) and (d) in function of the arm delay time δ or the correlation difference time $\xi=\langle i-k\rangle$. The cross-correlation function $G_{12}^{(2)}(0,\delta)$ reveals the famous HOM dip. It is imposed by the structure of the ultimate output state in the case of delta-correlated photons (σ→0)

$$\left|1,1\right\rangle_{out} = (T-R)\left|1,1\right\rangle + i\sqrt{2TR}\left|2,0\right\rangle + i\sqrt{2TR}\left|0,2\right\rangle. \quad \text{(S27)}$$

For a 50:50 BS, the probability amplitude to detect one photon at one output port and another photon in the other port given by the first term in this equation is exactly null.

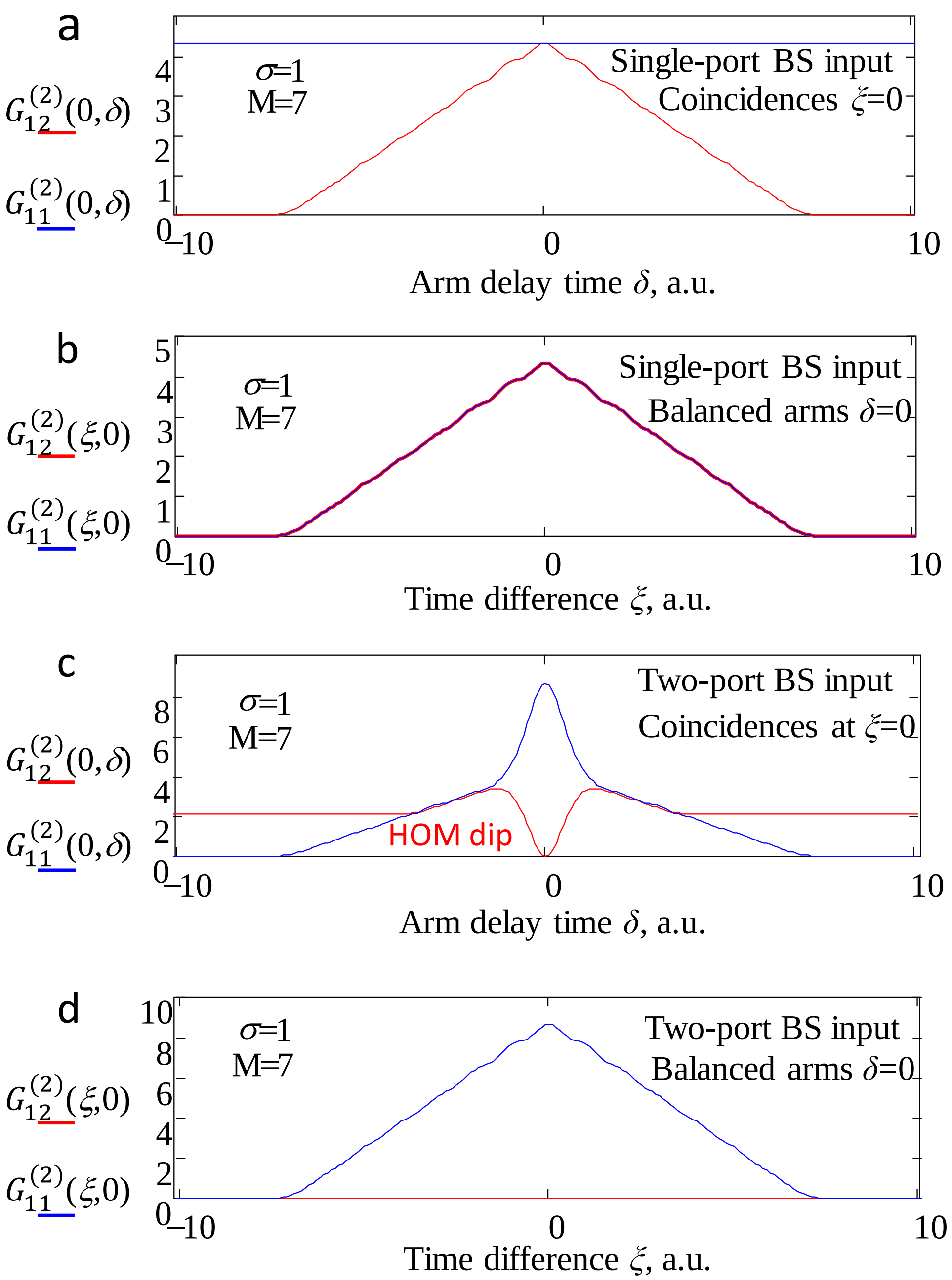

Figure S10. Correlation functions at BS output for single-port bi-photon state at the input [(a) and (b)] and for two-port input bi-photon state [(c) and (d)] modelled in the case of slow detectors. (a) and (c): joint coincidence rates (ξ=<i-k>=0) in function of the delay time δ between the output arms. (b) and (b): difference-time histograms at zero delay δ=0. Note HOM dip in the cross-correlation output trace (red line) in (c). The ideal beam splitter with T=R=50% is assumed. The detector response time M=7 exceeds the correlation time of the pair σ=1.

5 References

1. Gasparini, L., Bessire, B., Unternährer, M., Stefanov, A., Boiko, D., Perrenzoni, M. and Stoppa, D. Novel CMOS sensor for improved quantum imaging. *SPIE Newsroom, 11 April 2017,.* DOI: 10.1117/2.1201701.006847, 11 April 2017 (2017).
2. Gasparini, L., Bessire, B., Unternährer, M., Stefanov, A., Boiko, D., Perrenzoni, M. and Stoppa, D. SUPERTWIN: towards 100kpixel CMOS quantum image sensors for quantum optics applications. *SPIE Proceedings 10111, Quantum sensing and Nano Electronics and Photonics XIV,* 27 Jan 2017, 101112L, doi: 10.1117/12.2253598, (2017)
3. Lee, H. J, Kim, H. , Cha, M., and Moon, H. S., Generation of bright visible photon pairs using a periodically poled stoichiometric lithium tantalate crystal. *Optics Express* **23**, Issue 11, pp. 14203-14210; (2015).
4. Jabir M. V. and Samanta G. K., Robust, high brightness, degenerate entangled photon source at room temperature, Scientific Reports **7**, Article number 12613 (2017). https://doi.org/10.1364/OE.23.014203
5. Boiko, D. L., Gunther, , N. J., Brauer, N. , Sergio, M. , Niclass, C., Beretta, G. B.,and Charbon, E. A quantum imager for intensity correlated photons. *New J. Phys.* **11**, 013001–7 (2009).
6. Glauber, R. J. The Quantum Theory of Optical Coherence. *Phys. Rev.* **130**, 2529 (1963).
7. Glauber, R. J. Coherent and Incoherent States of the Radiation Field. *Phys. Rev.* **131** , 2766 (1963).
8. Niclass ,C., Rochas, A., Besse, P.-A., and Charbon, E. Design and characterization of a CMOS 3-D image sensor based on single photon avalanche diodes. *IEEE J. Solid State Circuits* **40**, no. 9, pp. 1847–1854, (2005).
9. Boiko, D. L., Gunther, N. J., Brauer, N., Sergio, M., Niclass, C., Beretta, G. B., and Charbon, E. On the application of a monolithic array for detecting intensity-correlated photons emitted by different source types. *Optics Express* **17**, Issue 17, pp. 15087-15103 (2009).
10. Saleh, B. E. A., Stoler, D., and Teich, M. C. Coherence and photon statistics for optical fields generated by Poisson random emissions. *Phys. Rev.* ***A*** **27**, 360 (1983).
11. D'Angelo, M., Chekhova, M. V., and Shih, Y. Two-Photon Diffraction and Quantum Lithography. *Phys. Rev. Lett.* **87**, 013602 (2001).
12. Ostermeyer, M., Puhlmann, D. and Korn, D. Quantum diffraction of biphotons at a blased grating. *J. Opt. Soc. Am.* ***B*****26** (No. 12), 2347-2356. (2009).
13. Shimizu, R., Edamatsu, K., Itoh, T. Quantum diffraction and interference of spatially correlated photon pairs generated by spontaneous parametric down-conversion. *Phys. Rev. A* **67**, 041805(R) (2003).
14. Law, C. K. and Eberly, J. H. Analysis and Interpretation of High Transverse Entanglement in Optical Parametric Down Conversion. *Phys. Rev. Lett.* **92**, 127903 (2004).
15. Feynman, R. P. *QED: The Strange Theory of Light and Matter*. Alix G. Mautner Memorial Lectures, Princeton University Press, Princeton, N.J., (1985)
16. Shih, Y. Quantum imaging. *IEEE J. Sel. Top. Quantum Electron.* **13**, 1016 (2007).
17. Agarwal, G.S. *Quantum Optics*, Cambridge University Press (2013).
18. Ghosh, R., and Mandel, L. Observation of Nonclassical Effects in the Interference of Two Photons. *Phys. Rev. Lett.* **59**, 1903 (1987).
19. Ou, Z.Y., Mandel, L., Observation of Spatial Quantum Beating with Separated Photodetectors. *Phys. Rev. Lett.* **61**, 54 (1988).
20. Hong, C. K., Ou, Z. Y., and Mandel, L. Measurement of subpicosecond time intervals between two photons by interference. *Phys. Rev. Lett.* **59** (18): 2044–2046, (1987).
